# Supplementary material for: Cognitive gripping with flexible graphene printed multi-sensor array
Source: Commun Eng. 2023 Aug 11;2:57. doi: 10.1038/s44172-023-00095-y (PMC11053132; doi:10.1038/s44172-023-00095-y)
Supplement: Supplementary file 2 — Supplementary Information [file 44172_2023_95_MOESM2_ESM.pdf]

Cognitive Gripping with Flexible Graphene Printed Multi-Sensor Array

Tania Mukherjee[1], Dipti Gupta[1]*

Abstract:

Robotics for task simplification of domestic, household, workplace and other assistive activities require efficient robots with decision-making capabilities. Here we report a fully printed graphene-based capacitive multi-sensor array (CAPSENSAR) employed in a cognitive robotic gripper (COGBOT) for decision-making operations. The CAPSENSAR created a contactless capacitive impression of the gripped object surface to determine the optimum gripping pressure. The controlling unit of the COGBOT was associated with an algorithm to address potential breakage. If slippage was detected via the array, the grip pressure was revised to reduce the possibility for damage. This facilitated slippage-free and damage-resistant gripping of the target objects without user interference. Array fabrication was straightforward using a customizable electrode design with cost-effective and biocompatible materials.

Key Words: Printed electronics, graphene- ink, proximity, sensor-array, cognitive gripper, robotics

[1] Plastic Electronics and Energy Laboratory (PEEL Lab), Metallurgical Engineering and Material Science, Indian Institute of Technology Bombay, Mumbai, India-400076. Email: diptig@iitb.ac.in

Supplementary sheet

Supplementary Discussion 1: Theory

The COGBOT constitutes a pair of CAPSENSARs and the robotic gripper which are connected electronically through the controlling unit. The proximity and pressure sensor array of the CAPSENSAR form the sensor and the monitoring unit while the gripper executes commands from the controlling unit. This section mathematically establishes the working principle of the proximity and pressure sensing abilities of the CAPSENSAR and henceforth the use of CAPSENSAR properties for cognitive gripping mechanism of the COGBOT.

Section 1.1: Mathematical Modelling of CAPSENSAR

The CAPSENSAR consists of mutually orthogonal arrangement of TE and BE at separate planes to form an arrangement of 5×4 capacitive proximity and pressure sensor array. The proximity sensor of the CAPSENSAR works on the principle of distortion in fringing electric field lines, which emanates from positively biased TE and terminates at negatively biased BE (Supp. Fig. 1), when an object is introduced in its vicinity. On the other hand, the pressure sensor works on the change in effective dielectric thickness of CAPSENSAR under applied pressure P. The device is mathematically investigated by analyzing the performance of an arbitrary sensor unit in the array. To investigate the performance of an arbitrary (i, j) elementary sensor unit in the CAPSENSAR, mathematical modelling was performed on that sensor unit by considering the influences due to the nearest and the second nearest neighboring sensor unit. Theoretical studies was carried out to determine the change in output capacitance ΔC experienced by the (i, j) sensor unit in presence of an approaching metallic object along the normal to that sensor unit at proximal distance z from it as discussed here.

Here we derive the expression for the change in output capacitance ΔC in a sensor unit due to the presence of an external metallic object. For this we consider an (3×3) array of sensor units representing a section of the CAPSENSAR as shown in Supp. Fig. 2. Each sensor unit, consisting of a TE and a BE, is schematically shown as square elements of side a and denoted by matrices (i, j), where (i, j) represents the central sensor unit, and $(i, j\pm1)$, $(i\pm1, j)$ and $(i\pm1, j\pm1)$ denotes the nearest and second nearest sensor units respectively. Since the TE is positively biased relative to the BE with an applied voltage V, the former holds a charge $+Q=\sigma_{\mathrm{TE}}.A$, while the later with the negative equivalent. Here, σ_{TE} denotes the surface charge density on TE and A is the effective area of the square shaped sub-electrodes sE. As the CAPSENSAR is designed with non-overlapping sE of TE and BE, the electric field lines emanating from the TE and terminating at the BE of the device are mostly due to fringing field effect of the TE, which are intense in the proximity of the electrodes and decays sharply with distance z. Here each identical sensor unit generates an intrinsic fringing electric field $E_{\mathrm{fr}}^{\mathrm{i,j}}(z)$ which spatially spreads over the neighboring space around the respective elements.

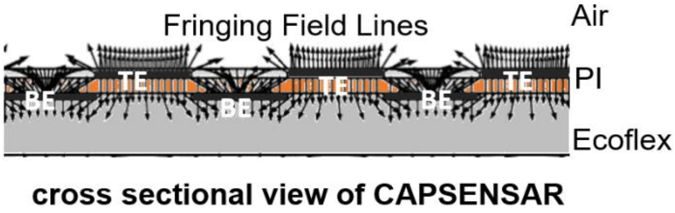

Supplementary Figure 1: Capacitive Fringing field lines of CAPSENSAR. Cross sectional view of the CAPSENSAR showing the electric fringing field lines emanating from the positively charged TE and converging in the negatively charged BE terminal.

A metallic object O is introduced at a distance z from the (i, j) sensor unit, it is immersed in the respective electric fields from each of the (3×3) representative array of sensor units, thereby experiencing a resultant field $E_{\text{fr}}^{\text{obj}}$. Thus $E_{\text{fr}}^{\text{obj}}$ is defined as the non-uniform fringing electric field between the object and the TE of the (i, j) sensor unit due to the charges Q_{obj} induced at the surface of the object by (3×3) sensor units. The contributions to $E_{\text{fr}}^{\text{obj}}$ includes (i) $E_{\text{fr}}^{\text{i,j}}(z)$ at a distance z from the (i, j) sensor unit, (ii) $E_{\text{fr}}^{(\text{i}\pm1,\text{j}),(\text{i},\text{j}\pm1)}(r)$ at a distance $r=\sqrt{z^2+\varsigma^2}$ from nearest neighbor sensor units, and (ii) $E_{\text{fr}}^{(\text{i}\pm1,\text{j}\pm1)}(r')$ at a distance $r'=\sqrt{z^2+2\varsigma^2}$ from second nearest neighbor sensor units, the total fringing field $E_{\text{fr}}^{\text{obj}}$ and expressed by:

$$E_{\text{fr}}^{\text{obj}}=E_{\text{fr}}^{\text{i,j}}(z)+E_{\text{fr}}^{(\text{i}\pm1,\text{j}),(\text{i},\text{j}\pm1)}(r)+E_{\text{fr}}^{(\text{i}\pm1,\text{j}\pm1)}(r') \text{ .... (Supp. Eq. 1),}$$

Thus, we first derive the expression of $E_{\text{fr}}^{\text{i,j}}(z)$ generated due to the contribution of σ_{TE} on the TE of (i, j) sensor unit. Let the area of an elementary charge unit on the TE of (i, j) sensor unit be area $dA=dx\times dy$ and the distance of the object at O from this elementary charge unit be R as shown in Supp. Fig. 2 (inset). The $E_{\text{fr}}^{\text{i,j}}(z)$ can be expresses as,

$$E_{\mathbf{fr}}^{\mathbf{i,j}}(z)=\iint dE_{\mathbf{fr}}^{\mathbf{i,j}}(z)\ \hat{\mathbf{k}}$$

$$=4\times\frac{1}{4\pi\varepsilon}\int_{y=0}^{a/2}\int_{x=0}^{a/2}\left(\frac{\sigma_{\mathbf{TE}}dA}{R^2}\times\cos\psi\right)\hat{\mathbf{k}}$$

$$=\frac{1}{\pi\varepsilon}\int_{y=0}^{a/2}\int_{x=0}^{a/2}\left(\frac{\sigma_{\mathbf{TE}}dxdy}{R^2}\times\cos\psi\right)\hat{\mathbf{k}} \text{ ....(Supp. Eq. 2a)}$$

where, the ψ and $\hat{\mathbf{k}}$ represent the angle between z and R and unit vector in z direction respectively. Substituting the $\cos\psi=\left(z/R\right)$ in Supp. Eq. 2a and using Supp. Fig. 2(inset), we get

$$E_{\mathbf{fr}}^{\mathbf{i,j}}(z)=\frac{\sigma_{\mathbf{TE}}}{\pi\varepsilon}\int_{y=0}^{a/2}\int_{x=0}^{a/2}\frac{z}{R^3}dxdy\ \hat{\mathbf{k}}$$

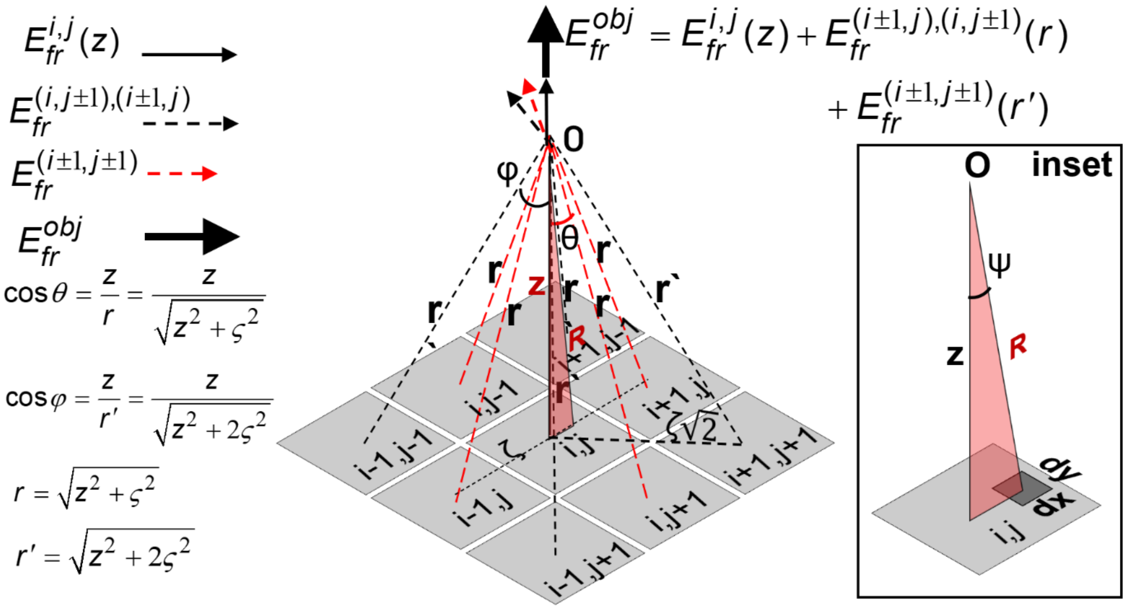

Supplementary Figure 2: Mathematical modelling of CAPSENSAR fringing field. Diagrammatic representation of (3×3) sensor unit array of a section of the CAPSENSAR showing the relative locations of sensor elements and the cumulative fringing field generated at the object *O* at normal distance *z* from (*i*×*j*) element.

$$= \frac{\sigma_{TE}}{\pi\varepsilon} \int_{y=0}^{a/2} \int_{x=0}^{a/2} \frac{z}{(x^2+y^2+z^2)^{3/2}} dxdy\ \hat{\mathbf{k}}$$

$$= \frac{\sigma_{TE}}{\pi\varepsilon} \int_{y=0}^{a/2} \left[\frac{zx}{(y^2+z^2)\sqrt{x^2+y^2+z^2}} \right]_0^{a/2} dy\ \hat{\mathbf{k}}$$

$$= \frac{\sigma_{TE}}{2\pi\varepsilon} \int_{y=0}^{a/2} \frac{(a \times x)}{(y^2+z^2)\sqrt{\frac{a^2}{4}+y^2+z^2}} dy\ \hat{\mathbf{k}}$$

$$= \frac{\sigma_{TE}}{2\pi\varepsilon} \times \left[2 \times \tan^{-1}\left(\frac{(a \times y)}{z\sqrt{a^2+4\times(y^2+z^2)}} \right) \right]_0^{a/2} \hat{\mathbf{k}}$$

$$= \frac{\sigma_{TE}}{\pi\varepsilon} \tan^{-1}\left(\frac{a^2}{2z\sqrt{2a^2+4z^2}} \right) \hat{\mathbf{k}} \quad \text{…………………………………………………………….(Supp. Eq. 2b)}$$

Thus, $E_{fr}^{(i,j\pm1),(i\pm1,j)} = \frac{\sigma_{TE}}{\pi\varepsilon} \tan^{-1}\left(\frac{a^2}{2z\sqrt{2a^2+4z^2}} \right)\left(\frac{z}{\sqrt{z^2+\zeta^2}} \right)$ ………………………………………..(Supp. Eq. 2c)

and $E_{fr}^{(i\pm1,j\pm1)} = \frac{\sigma_{TE}}{\pi\varepsilon} \tan^{-1}\left(\frac{a^2}{2z\sqrt{2a^2+4z^2}} \right)\left(\frac{z}{\sqrt{z^2+2\zeta^2}} \right)$ …………………..……………………….(Supp. Eq. 2d),

Where ζ represents the interelectrode distance of the TE array and ε denotes the absolute permittivity of the dielectric medium. Thus substituting Supp. Eq. 2b, c, d in Supp. Eq. (1) we get,

$$E_{\rm fr}^{\rm obj} = \frac{\sigma_{\rm TE}}{\pi\varepsilon}\tan^{-1}\left(\frac{a^2}{2z\sqrt{2a^2+4z^2}}\right)\left[1+4\left(\frac{z}{\sqrt{z^2+\zeta^2}}\right)+4\left(\frac{z}{\sqrt{z^2+2\zeta^2}}\right)\right] \text{..........................(Supp. Eq. 3)}$$

The $E_{\rm fr}^{\rm obj}$ acts along the normal to the plane of the (*i, j*) sensor unit and directed outward from point O. This presence of $E_{\rm fr}^{\rm obj}$ fringing lines of force produces distortion and annihilation of the $E_{\rm fr}^{\rm i,j}(z)$ fringing lines of force terminating at BE due to their interference at the proximity of the (*i, j*) sensor unit and produces a change in potential drop $\Delta V_{\rm i,j}$ across the TE and BE of the (*i, j*) sensor unit. The $\Delta V_{\rm i,j}$ occurs due to the presence of the object in the fringing electric field of the device and depends on the proximal distance $z >> d$ of the object from the (*i, j*) sensor unit, where *d* is the separation between the TE and the BE. The $\Delta V_{\rm i,j}$ expressed as $\Delta V_{\rm i,j} = \left(E_{\rm fr}^{\rm i,j} - E_{\rm fr}^{\rm obj}\right).z$ ....(Supp. Eq. 4).

In order to determine the change in capacitance ΔC incurred at an elementary sensor unit (*i, j*) of the CAPSENSAR when an metallic object O is introduced at a distance *z* normal to (*i, j*) sensor unit, we ascertain the charges present on the TE, BE after the introduction of the metallic object O. Now, the positive charge $+Q = \sigma_{\rm TE}.A$ on TE induces charge $-Q_{\rm obj}$ on the surface of the object such that:

$$+Q = Q_{\rm obj} + Q_{\rm BE} \text{ .... (Supp. Eq. 5),}$$

where, $-Q_{\rm BE}$ denotes the charge on BE. Thus putting the values of $Q_{\rm obj} = \sigma_{\rm obj}.S$ and $Q_{\rm BE} = \sigma_{\rm BE}.A$ in Supp. Eq. (5) and rearranging we have: $(\sigma_{\rm TE} - \sigma_{\rm BE}).A = \sigma_{\rm obj}.S$, ...(Supp. Eq. 6)

Where $\sigma_{\rm obj}$ and $\sigma_{\rm BE}$ represents the surface charge density on the surface of object and BE respectively and S denotes the surface area of the object exposed to the (*i,j*) sensor unit. Since *A* is a constant and $\sigma_{\rm obj}$ is a material property and depends on the metal object used in the investigation, thus $(\sigma_{\rm TE} - \sigma_{\rm BE})$ is a metal dependent quantity. If we assume that $\left.C_{\rm out}^{\rm i,j}\right]^{\rm abs}$ is the intrinsic output capacitance between the TE and BE of the (*i, j*) sensor unit and $\left.C_{\rm out}^{\rm i,j}\right]^{\rm pre}$ is the output capacitance as recorded by the same sensor unit in presence of the object, then the spatial location of object in terms of the proximal distance *z* may be determined by measuring $\Delta C_{\rm PROX} = \left.C_{\rm out}^{\rm i,j}\right]^{\rm abs} - \left.C_{\rm out}^{\rm i,j}\right]^{\rm pre}$ ......(Supp. Eq. 7)

The ΔC can be theoretically expressed using Supp. Eq. (4) and Supp. Eq. (6) as:

$$\Delta C_{\rm PROX} = \frac{Q_{\rm obj}}{-\Delta V_{\rm i,j}} = \frac{(\sigma_{\rm TE} - \sigma_{\rm BE}).A}{\left(E_{\rm fr}^{\rm i,j} - E_{\rm fr}^{\rm obj}\right).z} \text{ ....(Supp. Eq. 8)}$$

Where, $\Delta V_{\rm i,j}$ denotes the change in potential drop across the TE and BE of the (*i, j*) sensor unit, $E_{\rm fr}^{\rm obj}$ is the non-uniform fringing electric field between the object and the TE due to the charges $Q_{\rm obj}$ induced at the surface of the object and has an arctan dependence with *z* (Supp. Fig. 3a), $E_{\rm fr}^{\rm i,j}$ intrinsic electric field between TE and BE when the object is absent , σ_{TE} and σ_{BE} are surface charge densities of TE and BE electrodes respectively and $(\sigma_{\rm TE} - \sigma_{\rm BE}).A$ is material

dependent quantity since $(\sigma_{\mathrm{TE}} - \sigma_{\mathrm{BE}}).A = \sigma_{\mathrm{obj}}.S$, where σ_{obj} denotes the surface charge density on the object of surface area S, A effective area of a sensor unit of side a. For any z, since $E_{\mathrm{fr}}^{\mathrm{i,j}} > E_{\mathrm{fr}}^{\mathrm{obj}}$, the $\left.C_{\mathrm{out}}^{\mathrm{i,j}}\right]^{\mathrm{pre}} < \left.\mathrm{C}_{\mathrm{out}}^{\mathrm{i,j}}\right]^{\mathrm{abs}}$ according to Supp. Eq. (7) and Supp. Eq. (8). The $-\Delta V_{\mathrm{i,j}}$ is indicative of the reduction in potential drop between TE and BE, resulting in the decrease in the $\left.C_{\mathrm{out}}^{\mathrm{i,j}}\right]^{\mathrm{pre}}$ in presence of the object. The change in output capacitance ΔC_{PROX} of the (i, j) proximity sensor unit is calculated by considering non-overlapping square geometry of sE of TE and BE in parallel capacitive arrangement and obtained by substituting the value of $E_{\mathrm{fr}}^{\mathrm{obj}}$ from Supp. Eq. (3) in Supp. Eq. (8) we get:

$$\Delta C_{\mathrm{PROX}} = \frac{(\sigma_{\mathrm{TE}} - \sigma_{\mathrm{BE}}).A}{z.\left[E_{\mathrm{fr}}^{\mathrm{i,j}} - \frac{\sigma_{\mathrm{TE}}}{\pi\varepsilon}\tan^{-1}\left(\frac{a^2}{2\sqrt{2}z\sqrt{a^2+2z^2}}\right)\left[1+4\left(\frac{z}{\sqrt{z^2+\zeta^2}}\right)+4\left(\frac{z}{\sqrt{z^2+2\zeta^2}}\right)\right]\right]} \quad \text{………... (Supp. Eq. 9)}$$

where ε and ς are the absolute dielectric permittivity of the medium (air) and interelectrode distance between two sub electrodes of adjacent TEs respectively.

The ΔC_{PROX} increases sharply when the object approaches $z \to 0$, while the function decays to zero at large $z \to \infty$ to yield $\left.C_{\mathrm{out}}^{\mathrm{i,j}}\right]^{\mathrm{pre}} \to \left.C_{\mathrm{out}}^{\mathrm{i,j}}\right]^{\mathrm{abs}}$. Since the charge density on the surface of a metallic object (conductor) with finite conductivity depends on the skin depth of the metal at a fixed frequency, the σ_{obj} is dependent on the metallic property of the object. Thus the ΔC_{PROX} varies when the experiment is performed with object of different metal having same shape and size. Although the σ_{obj} is a shape dependent quantity, the ΔC_{PROX} of the single sensor unit is affected when the dimension of the local curvature<< a=3 mm i.e sharp. Thus for objects of dimensions $\geq a$, exposed to the sensor unit at a fixed z, may be considered as planar for which ΔC_{PROX} is independent of shape and size of the object and solely depends on z. However, for an array of sensor units, each sensor unit records their respective ΔC_{PROX} to construct a capacitive landscape of the segmented face of the object. The dimensions and surface morphology variations of the object can be computed from the calibration curve of the proximity sensor array. Thus, metallic objects (with dimensions 30 × 24 mm) of various shapes and sizes can be distinguished from capacitive impressions generated by the CAPSENSAR. On the contrary, when a dielectric material of permittivity ε_{r} is introduced at a proximal distance z from the (i, j) sensor unit, the $\left.C_{\mathrm{out}}^{\mathrm{i,j}}\right]^{\mathrm{pre}} > \left.\mathrm{C}_{\mathrm{out}}^{\mathrm{i,j}}\right]^{\mathrm{abs}}$ as the effective dielectric thickness of the device decreases (see Supp. Disc. 1.2).

At z=0 (touch) when the metallic object touches the TE of the sensor unit, the capacitance between TE and the object is annulled and the $\left.C_{\mathrm{out}}^{\mathrm{i,j}}\right]^{\mathrm{pre}}$ reduces to: $\left.C_{\mathrm{out}}^{\mathrm{i,j}}\right]_{z\to 0}^{\mathrm{pre}} \left.C_{\mathrm{out}}^{\mathrm{i,j}}\right]^{\mathrm{abs}} - \max|\Delta C_{\mathrm{PROX}}|$ ………………….(Supp. Eq. 10),

where $\max|\Delta C_{\mathrm{PROX}}| \neq \infty$ is a constant for a fixed material and obtained due to roughness of object surface in contact

with the TE. Under this condition, the pressure sensor unit records the applied P which commences from the pressure sensing baseline as given by $\left.C_{\text{out}}^{\text{i,j}}\right]_{z\to 0}^{\text{pre}}$ in Supp. Eq. (10), where $\left.C_{\text{out}}^{\text{i,j}}\right]^{\text{abs}} = \dfrac{\sigma_{\text{TE}} A}{E_{\text{fr}}^{\text{i,j}}.d_{\text{fr}}\,|_{z=0}}$ ….......Supp. Eq. (11) and

d_{fr} is the effective dielectric thickness of the device in the presence of the object at z=0. The $\left.C_{\text{out}}^{\text{i,j}}\right]_{z\to 0}^{\text{pre}}$ is a constant quantity for a particular object, obtained under just contact condition z=0. The effective dielectric layer constitutes the parallelly arranged Eco-flex and PI of thicknesses d_{PI} and δ respectively. Under applied pressure P, the decrease in thickness of the eco-flex elastomeric dielectric layer reduces the d_{fr} which linearly increases the $\left.C_{\text{out}}^{\text{i,j}}\right]^{\text{pre}}$ with applied pressure P on the sensor unit as:

$$\left.C_{\text{out}}^{\text{i,j}}\right]^{\text{pre}} = \frac{\sigma_{\text{TE}} A.P}{E_{\text{fr}}^{\text{i,j}}.K} - \max\left|\Delta C_{\text{PROX}}\right|, \quad \text{………………………... (Supp. Eq. 12)}$$

$$\text{i.e } \Delta C_{\text{PRES}} = \left.C_{\text{out}}^{\text{i,j}}\right]^{\text{pre}} - \left.C_{\text{out}}^{\text{i,j}}\right]_{z\to 0}^{\text{pre}} = \frac{\sigma_{\text{TE}} A.P}{E_{\text{fr}}^{\text{i,j}}.K} - \frac{\sigma_{\text{TE}} A}{E_{\text{fr}}^{\text{i,j}}.d_{\text{fr}}\,|_{z=0}} \quad \text{……..……. (Supp. Eq. 13)}$$

where $d_{\text{fr}} = K.P^{-1}$ and K proportionality constant. The ΔC_{PRES} increases linearly with pressure P with slope $\dfrac{\sigma_{\text{TE}} A}{E_{\text{fr}}^{\text{i,j}}.K}$ as the second term in (Supp. Eq. 13) is a constant for a fixed object.

Section 1.2: Effect of dielectric target object on output capacitance

Now we determine the change in output capacitance of the (i, j) sensor unit due to an approaching insulating object at proximal distance z. Here we consider a dielectric object of thickness τ, relative dielectric constant ε_{r} approaching the (i, j) sensor unit along its normal direction of (-z). The output capacitance measured by the (i, j) sensor unit is given by $\left.C_{\text{out}}^{\text{i,j}}\right] = \dfrac{Q}{V_{\text{i,j}}}$, where Q is the charge on TE and $V_{i,j}$ is the potential drop across TE and BE. In the absence of the object, the output capacitance is given by $\left.C_{\text{out}}^{\text{i,j}}\right]^{\text{abs}} = \dfrac{Q}{E_{\text{fr}}^{\text{i,j}}.d_{\text{fr}}(z)}$. When the dielectric object is exposed to the (i, j) sensor unit of the CAPSENSAR in air of $\varepsilon_{\text{r}} = 1$, the output capacitance $\left.C_{\text{out}}^{\text{i,j}}\right]_{\text{die}}^{\text{pre}} = \dfrac{Q}{V_{\text{i,j}}}$ as the potential drop $V_{\text{i,j}}$ across TE and BE decreases. The $V_{\text{i,j}}$ between TE and BE in presence of the object is calculated using the relation: $V_{\text{i,j}} = \left(d_{\text{fr}}(z) - \tau\right) E_{\text{fr}}^{\text{i,j}} + \dfrac{E_{\text{fr}}^{\text{i,j}}}{\varepsilon_{\text{r}}}\tau$, where $E_{\text{fr}}^{\text{i,j}}$ is the intrinsic fringing field between TE and BE and $d_{\text{fr}}(z) = \Theta.z$ is the effective dielectric thickness of the sensor unit for z>0, where Θ is the linear proportionality function of z.

$$\text{Thus } V_{\text{i,j}} = \left(\Theta.z - \tau\right) E_{\text{fr}}^{\text{i,j}} + \frac{E_{\text{fr}}^{\text{i,j}}}{\varepsilon_{\text{r}}}\tau$$

$$V_{\mathrm{i,j}} = \left(\Theta.z - \tau\right) E_{\mathrm{fr}}^{\mathrm{i,j}} + \frac{E_{\mathrm{fr}}^{\mathrm{i,j}}}{\varepsilon_{\mathrm{r}}} \tau$$

$$V_{\mathrm{i,j}} = E_{\mathrm{fr}}^{\mathrm{i,j}} \left[\Theta.z - \tau + \frac{\tau}{\varepsilon_{\mathrm{r}}} \right]$$

$$V_{\mathrm{i,j}} = E_{\mathrm{fr}}^{\mathrm{i,j}} \left[\Theta.z - \tau \left(1 + \frac{1}{\varepsilon_{\mathrm{r}}} \right) \right]$$

Output capacitance of the (*i*,*j*) sensor unit in presence of the object is given by

$$\left. C_{\mathrm{out}}^{\mathrm{i,j}} \right]_{\mathrm{die}}^{\mathrm{pre}} = \frac{Q}{V_{\mathrm{i,j}}} = \frac{\sigma_{\mathrm{TE}}.A}{E_{\mathrm{fr}}^{\mathrm{i,j}} \left[\Theta.z - \tau \left(1 + \frac{1}{\varepsilon_{\mathrm{r}}} \right) \right]} \quad \text{.....................(Supp. Eq. 14)}$$

Unlike metallic object, when a dielectric material of permittivity ε_r is introduced at a proximal distance z from the (*i*,*j*) sensor unit, the $\left. C_{\mathrm{out}}^{\mathrm{i,j}} \right]^{\mathrm{pre}} = \frac{Q}{E_{\mathrm{fr}}^{\mathrm{i,j}} \left[\Theta.z - \tau \left(1 + \frac{1}{\varepsilon_{\mathrm{r}}} \right) \right]}$ increases relative to $\left. C_{\mathrm{out}}^{\mathrm{i,j}} \right]^{\mathrm{abs}} \left(= \frac{Q}{E_{\mathrm{fr}}^{\mathrm{i,j}}.\Theta.z} \right)$ as the $\Gamma(z)$ decreases by $\tau \left(1 + \frac{1}{\varepsilon_{\mathrm{r}}} \right)$ on introduction of the dielectric object. For a fixed object, the $\left. C_{\mathrm{out}}^{\mathrm{i,j}} \right]^{\mathrm{pre}}$ varies linearly with *z*.

Section 1.3: Operation of the Cognitive robotic (COGBOT) gripper

The CAPSENSAR (L, R) integrated on the gripper palm (L, R) of the COGBOT is useful for cognitive gripping of the target object and also ensuring slippage and deformation free gripping. Since strong grip is associated with large gripping area, the COGBOT is programmed to determine the pair of faces of the target object with flattest area for effective gripping. The pair of CAPSENSAR using the proximity sensor array creates electronic capacitive impression of different faces of the target object as the arrangement of the pair of robotic palms with CAPSENSARs rotates about the central axis passing through object, thereby scanning the object faces through 360°. The capacitive impressions of different faces of the objects were transformed to their respective proximity distance *z*-equivalent matrices to estimate the landscape of each of the scanned object faces and their dimensions. The COGBOT performs cognitive computational operations to analyze these z-matrices to determine the fittest pair of opposite gripping faces with largest flat area for effective gripping. The identification of pair of flattest face for gripping allows the COGBOT, to grip the given object with optimum pressure to prevent damage. The optimum pressure was determined using computational steps and contributes to the cognitive operation of the COGBOT. The pressure sensor array of CAPSENSAR initially captures the capacitive impression of the object faces under gripped condition and transform the same into its equivalent *P*-matrices for the pair of opposite faces of the gripped object. The *P*-matrices help to ensure that the optimum pressure for gripping is maintained throughout the operation. The *P*-matrices are also used for the detection of slippage of gripped object and also monitor deformation of the object during gripping.

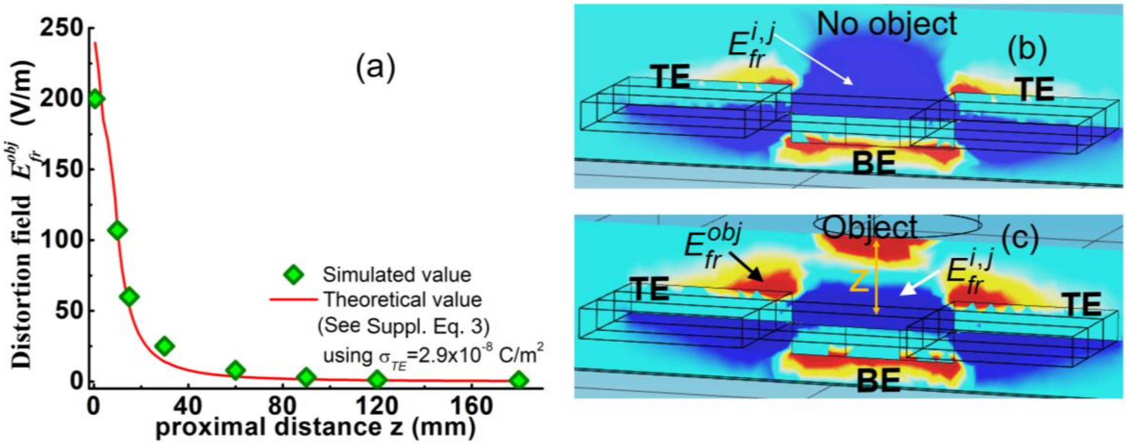

Supplementary Figure 3: Simulated fringing electric field distribution near the sensor unit. a. Variation of simulated $E_{\text{fr}}^{\text{obj}}$ with proximal distance *z*. The green rhombus data points were acquired from simulation investigation shown in supplementary Fig. 4. The red solid line denotes the theoretical arctan relation obtained from supplementary Eq. 3. COMSOL representation of fringing electric field $E_{\text{fr}}^{\text{i,j}}$ and $E_{\text{fr}}^{\text{obj}}$ distribution around TE and BE of the (i, j) sensor unit in the **b.** absence and **c.** presence of object at proximal distance *z.*

Supplementary Discussion 2: Simulation Results

As explained in Supp. Disc. 1, the CAPSENSAR operates on the principle of variation of fringing field capacitance for the measurement of (a) proximal distance *z* of the object from the device and thereby providing a capacitive impression of face landscape and (b) the force exerted on the device by the object during gripping. Since the fringing electric field are predominant at the non-overlapping region, the CAPSENSAR is designed with capacitive architecture with non-overlapping TE and BE to maximize the fringing electric field at the proximity of the device surface. The simulation studies were performed to investigate the role of the non-overlapping area between the sub-electrodes sE of TE and the BE. We investigate the spatial electric field due to the square geometry of sub electrode design and hence optimize the non-overlapping area to increase the sensitivity and dynamic range of the CAPSENSAR. The working of the CAPSENSAR is demonstrated through simulation studies with TE positively biased relative to BE using a $(i, j)^{\text{th}}$ elementary sensor unit.

Section 2.1*:* Proximity sensing

The determination of the *z* of the target object (with diameter ~*a*) from the $(i, j)^{\text{th}}$ sensor unit of the CAPSENSAR was obtained by measuring the variation in the fringing field capacitance ΔC_{PROX} between TE and BE. The ΔC_{PROX} occurs due to the variation in spatial fringing electric field distribution $\Delta E_{\text{fr}}^{\text{i,j}} = E_{\text{fr}}^{\text{i,j}} - E_{\text{fr}}^{\text{obj}}$ due to an approaching object at *z* in the neighborhood of the $(i, j)^{\text{th}}$ sensor unit. The effect of the variation in $E_{\text{fr}}^{\text{i,j}}$ distribution due to the object near the $(i, j)^{\text{th}}$ sensor unit was determined by considering $E_{\text{fr}}^{\text{obj}}$ generated near the device. Theoretical results obtained

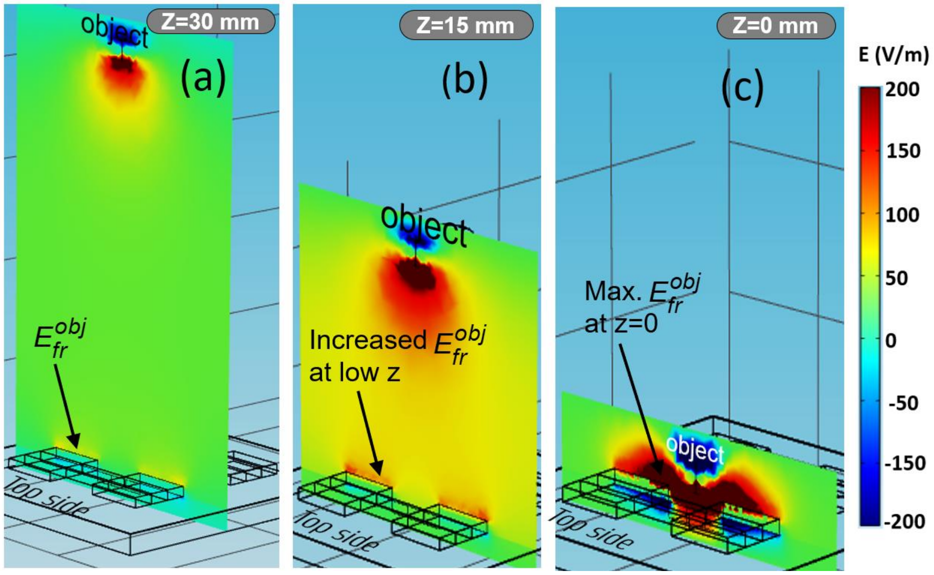

Supplementary Figure 4: Simulation studies with proximity sensor unit. Fringing electric field distribution at different proximal distances **a.** *z*=30 mm, **b.** *z*=15 mm and **c.** in contact *z*=0 mm

from (Supp Disc. 1) showed that for an approaching object the $E_{\mathrm{fr}}^{\mathrm{obj}}$ increases as *z* reduces obeying the arctan relation as shown in Supp. Fig. 3a. Within close proximity of the device the $E_{\mathrm{fr}}^{\mathrm{obj}}$ sharply increases as $z \to 0$. As the object approaches the $(i, j)^{\mathrm{th}}$ sensor unit , the distortion field $E_{\mathrm{fr}}^{\mathrm{obj}}$ interfere with the intrinsic $E_{\mathrm{fr}}^{\mathrm{i,j}}$ distribution of the device to produce a change in the effective $E_{\mathrm{fr}}^{\mathrm{i,j}}$ at the that sensor unit as shown in Supp. Fig. 3b, c. The spatial distribution of the distortion field $E_{\mathrm{fr}}^{\mathrm{obj}}$ at TE of the $(i, j)^{\mathrm{th}}$ sensor unit for object at for *z*= 30 , 15 and 0 mm are illustrated in Supp. Fig. 4a, b and c respectively. This distortion field $E_{\mathrm{fr}}^{\mathrm{obj}} \to 0$ at large distances and becomes high $E_{\mathrm{fr}}^{\mathrm{obj}}$ = 200 Vm^{-1} as $z \to 0$. The values of $E_{\mathrm{fr}}^{\mathrm{obj}}$ for object placed at different *z* in the range *z*=0-180 mm was obtained from simulation results and was compared with that of the theoretical counterpart as shown in Supp. Fig. 3a. The excellent match between the simulation and the theoretical results was indicative of a dependence of $E_{\mathrm{fr}}^{\mathrm{obj}}$ on arctan (*z*) owing to the non-overlapping square sE architecture of TE and BE which offer non-uniform fringing electric field in proximal space. The increased distortion field $E_{\mathrm{fr}}^{\mathrm{obj}}$ at reduced *z*, increases the $\Delta E_{\mathrm{fr}}^{\mathrm{i,j}}$ and hence the $\Delta C_{\mathrm{PROX}} = \dfrac{Q_{\mathrm{obj}}}{\Delta E_{\mathrm{fr}}^{\mathrm{i,j}}.z}$. Thus, proximal distance *z* of the approaching object can be measured in terms of ΔC_{PROX}. The capacitive architecture of non- overlapping sub electrodes of TE and BE, with optimized distance between adjacent electrodes ς in an array, yield a detectable proximal distance of *z*=120 mm. An array of such $(i \times j)$ identical sensor units operated separately to create a capacitive impression and hence the *z*-landscape of the exposed object face as discussed below. The non-uniform fringing electric field spanned over large *z* in space facilitates non-contact shape detection of distant object.

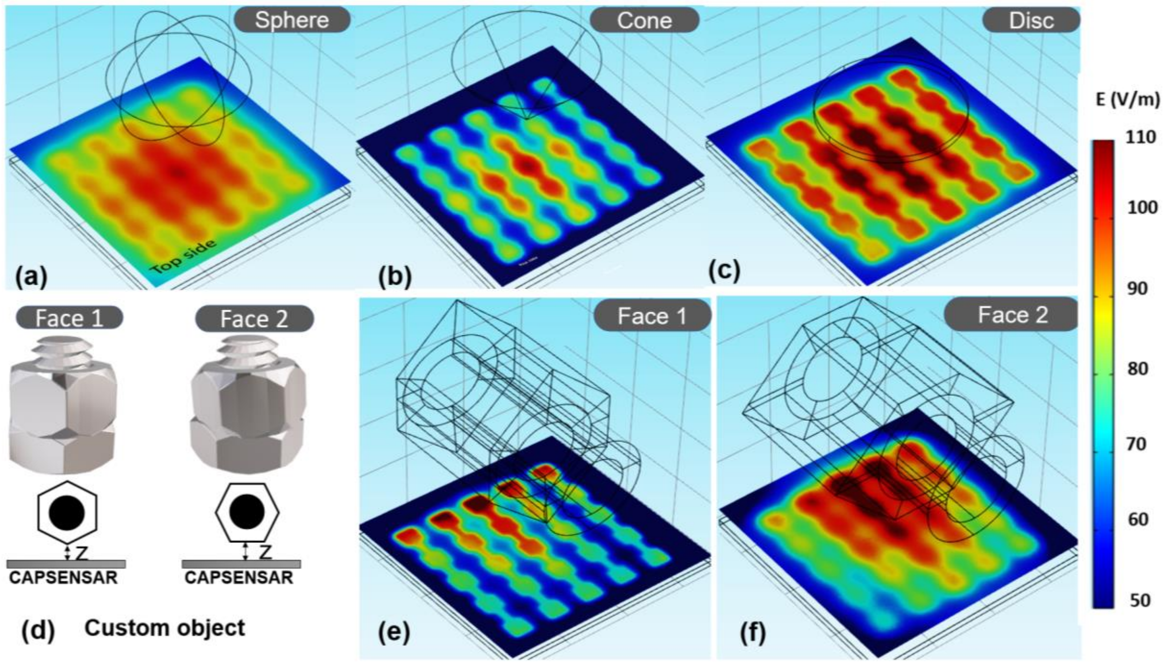

Supplementary Figure 5: Simulation studies on face landscape detection. COMSOL representation of the fringing electric field distribution (E_{fr} array impression) on the elementary sensor units of the CAPSENSAR under exposure to different solid objects –**a.** sphere, **b.** cone **c.** disc and for **d.** Illustration of different exposure faces- Face 1 and Face 2 of the custom-made test object. COMSOL simulation results for E_{fr} array impression captured by the CAPSENSAR when **e.** Face 1 and **f.** Face 2 of the object were placed at distance z=10 mm from the device.

Section 2.2: Face landscape estimation

The face landscape estimation of a given object was achieved using $(i\times j)$ proximity sensor array of the CAPSENSAR where each (i, j) elementary sensor unit measured respective $\left.C_{\mathrm{out}}^{\mathrm{i,j}}\right]^{\mathrm{pre}}$ based on the projective proximal distance z of the (i, j) sensor unit from the segmented landscape of the exposed face of the object, thereby creating an $(i\times j)$ array of capacitive impression of the face of the object. Since the measurable $\left.C_{\mathrm{out}}^{\mathrm{i,j}}\right]^{\mathrm{pre}}$ is a strong function of the distortion field $E_{\mathrm{fr}}^{\mathrm{obj}}$ (following Supp. Eq. (7) and Supp. Eq. (8)), the face landscape simulations were investigated in terms of $E_{\mathrm{fr}}^{\mathrm{obj}}$ generated separately at different (i, j) sensor units. The sensor units which are at low z from segmented exposed face of the object generated high $E_{\mathrm{fr}}^{\mathrm{obj}}$ relative to that which are comparatively at furthest distances. The $(i\times j)$ array impression of $E_{\mathrm{fr}}^{\mathrm{obj}}$ generated from the exposed object face facilitated recognition of face landscape and even determination of the dimension of the object. The simulation studies of face landscape recognition were investigated using various geometric shaped objects (with dimensions ~ i and j =20 mm) such as steel sphere, cone and disc kept at z=10 mm from the CAPSENSAR. Supp. Fig. 5a, b and c show the $E_{\mathrm{fr}}^{\mathrm{obj}}$ -array impression for various geometric

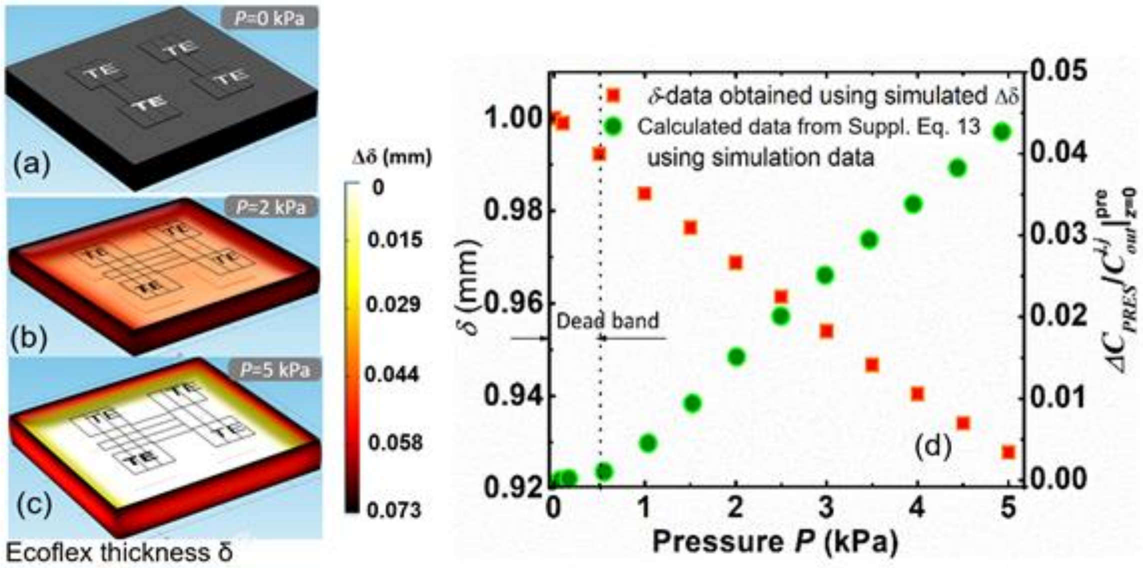

Supplementary Figure 6: Simulation studies with pressure sensor unit. COMSOL representation of Eco-flex thickness variation $\Delta\delta$ for **a.** *P*= 0 kPa, **b.** 2 kPa, **c.** 5 kPa and **d.** Variation of Ecoflex thickness and its corresponding change in normalized output capacitance with applied pressure *P*. The red solid square data points represent simulated Ecoflex thickness and the green solid circle denotes data points obtained from supplementary Eq. 13.

shapes-sphere, cone and disc respectively. It is evident from Supp. Fig. 5a-c that the lateral variation in $E_{\mathrm{fr}}^{\mathrm{obj}}$ in the *x*- and *y*-direction is clearly distinguishable for objects of different shapes and that these $E_{\mathrm{fr}}^{\mathrm{obj}}$ -array impressions bear strong manifestations of 3-dimensional landscape of respective object faces. The stimulations were also performed by exposing different faces of the same custom-made object to the CAPSENSAR as illustrated in Supp. Fig. 5d When Face 1 and Face 2 of this custom-made object were exposed separately to the CAPSENSAR, the $E_{\mathrm{fr}}^{\mathrm{obj}}$ array impressions for respective faces yielded distinctive patterns as shown in Supp. Fig. 5e and Supp. Fig. 5f respectively. The Face 2 generated a large flatter area as compared to Face 1 where the former face was suitable for reliable gripping. The face landscape estimation ability of the CAPSENSAR is utilized in COGBOT in the identification of flattest pair of opposite faces of the object which is useful in calculating the optimized gripping area and execute successful gripping.

Section 2.3: Pressure sensing

The simulation studies were carried out to investigate the effect of applied pressure *P* on the effective dielectric thickness d_{fr} in the $(i, j)^{\mathrm{th}}$ elementary sensor unit of CAPAENSAR. When the object was in contact with the device *z*=0, the effective dielectric thickness $d_{\mathrm{fr}}\,|_{z=0}$ incorporates the PI thickness d_{PI} and Ecoflex thickness δ . Since the d_{PI} is constant, the application of an external *P* by the object on the $(i, j)^{\mathrm{th}}$ sensor unit produced a change $\Delta\delta$ in thickness of the elastomeric Eco-flex dielectric layer. Simulation studies were performed to determine the displacement $\Delta\delta$ in the Ecoflex layer of $(i, j)^{\mathrm{th}}$ sensor unit under different applied *P* in the range 0-5 kPa. The $\Delta\delta$ occurs in the -z direction

in terms of the position of the object on the device (z=0). Supp. Fig. 6a, b and c show the COMSOL representation illustrating the $\Delta\delta$ in Ecoflex thickness under P=0 kPa, 2 kPa and 5 kPa respectively. At increased pressure P, the Ecoflex thickness δ was reduced, which led to the increase in ΔC_{PROX} of the device following Supp. Eq. (13). The linear variation of δ and ΔC_{PROX} with P as obtained from simulation results are plotted in Supp. Fig. 6d. The variation in output capacitance due to change in δ under applied pressure was utilized to measure the gripping force to be applied on the target object.

Supplementary Discussion 3: Fabrication

Section 3.1: Materials and equipment

High temperature heat resistant polyimide (PI) tape (Kapton Tape) of thickness 30 µm and Size (24 mm ×30 m) for Transfer Printing, Smooth Ecoflex 00-30 Soft Silicone Liquid Rubber and 3D Printing Material ABS 3D Printing Filament (Red) with Print temperature- 220-230 °C were purchased from Amazon. Graphene ink (793663-5ML) of resistivity 0.003-0.008 Ω cm and Poly (vinyl alcohol) (341584-25G) of Mw 89,000-98,000, 99% hydrolyzed were purchased from Sigma Aldrich, USA. 3D printing of models was implemented using Ultimaker 2+Connect. Graphene printing of electrodes were performed using Jetlab® 4 - Tabletop Printing Platform, MicroFab Technologies, Inc., USA

Section 3.2: Fabrication of CAPSENSAR

The device was fabricated in six steps as shown in Supp. Fig. 7. The polyimide (PI) sheet was properly cleaned and made hydrophilic by using the O^{2-} plasma to achieve the good printability for graphene ink of viscosity=8 mPa.s. Then this low viscous printable graphene ink was inkjet printed on the processed surface of PI to realize the 200 nm thick top electrodes (TEs) as shown in Supp. Fig. 7a Inset 1. The 300 S cm^{-1} high conductivity of the TEs was achieved by heating the printed graphene layer in the vacuum oven at a temperature (T) of 250 °C for 30 min. Supp. Fig. 7a Inset 2 shows that the non-contact ink-jet printing method (using print head of diameter=30 µm) was mask-less and material wastage free and is fast with print throughput of 15 min and printing rate 20 mm s^{-1}. In Supp. Fig. 7b, the TEs were masked by spray coating the Polyvinyl alcohol (PVA) followed by its curing at T=60 °C. In Supp. Fig. 7c, the PI sheet was flipped to make its reverse side exposed for bottom electrodes (BE) printing. Then, this exposed PI side was prepared for BE printing by making it clean and hydrophilic in the similar method as discussed in Supp. Fig. 7a. The graphene based BEs were inkjet printed on the processed surface of the reverse side of PI following technique shown in Supp. Fig. 7a, and dried in vacuum oven at a low temperature of 60°C as shown in Supp. Fig. 7d. Then the PVA masking layer on the TE was removed by rinsing it using DI water followed by the heating of BEs in the vacuum oven at a temperature (T) of 250 °C for 30 min (Supp. Fig. 7e) to achieve their high conductivity as discussed in step (a).

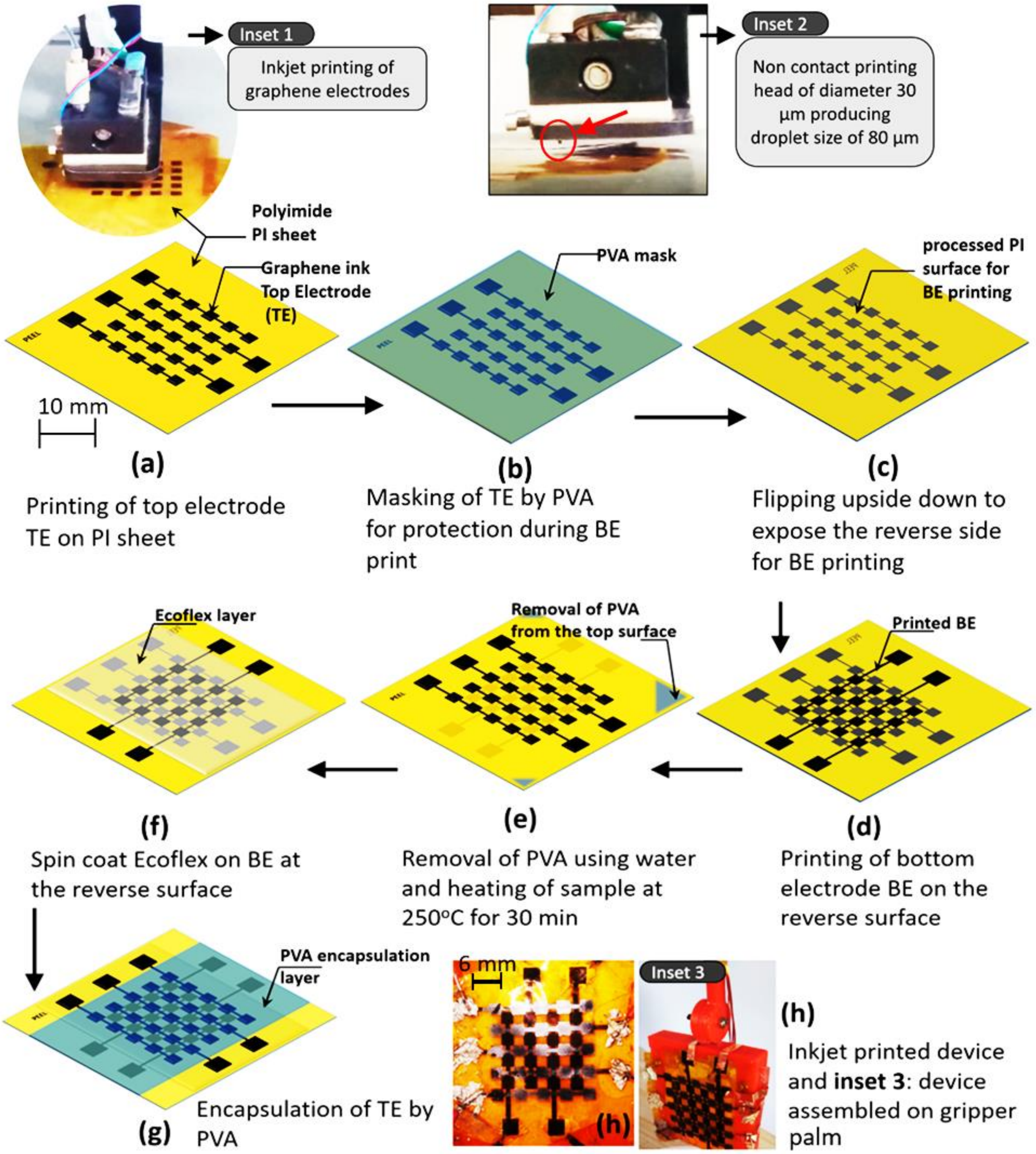

Supplementary Figure 7: Schematic representation of fabrication process flow–a. Printing of TE on PI, (Inset I) Ink-jet printing of graphene ink on PI and (Inset 2) Noncontact and mask-less printing of graphene electrodes on PI **b.** Masking of TE by PVA for protection during BE print, **c.** Flipping upside down to expose the reverse side of BE printing, **d.** Printing of BE on the reverse side, **e.** removal of PVA using water and heating the sample, **f.** Spin coat Eco-flex on BE at the reverse side, **g.** encapsulation of TE by PVA and **h.** Image of CAPSENSAR and (Inset 3) Image of CAPSENSAR integrated on the palm of COGBOT

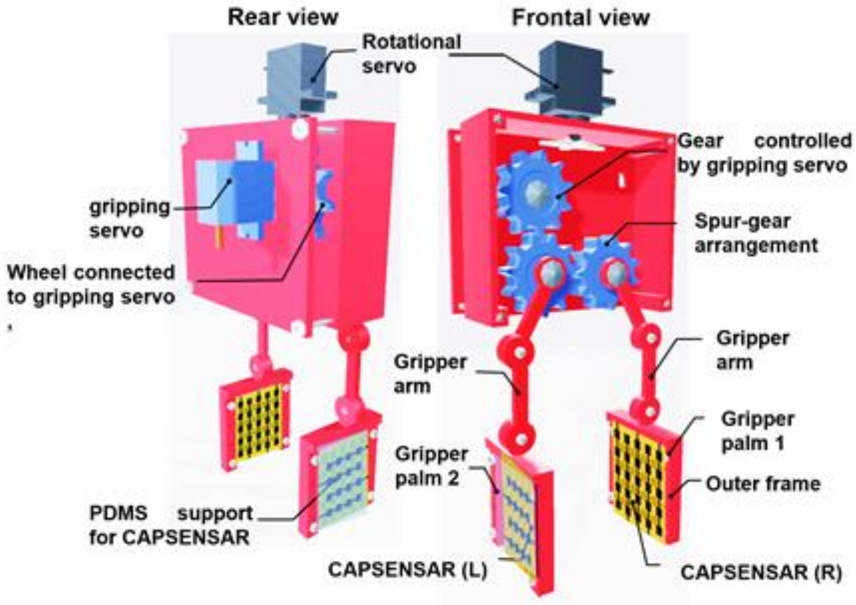

Supplementary Figure 8: Components of COGBOT. Schematic diagram of COGBOT showing its rear and frontal view and primarily comprise of the rotational servo capable of rotation in the range 0-150º for aligning the pair of gripping palms in appropriate orientation for effective gripping, the spur gear arrangement supported by gripping servo capable of rotation in the range 0-20º for gripping and release and a pair of CAPSENSAR (R,L) attached onto gripping palms 1,2

Now the rear side cladding of the device was realized by spin coating the eco-flex solution on the BE at a rotation speed of 500 rms and subsequently, cured it at T=60 °C as shown in Supp. Fig. 7f. In Supp. Fig. 7g, the device is again flipped to passivate its top electrodes by spray coating the PVA layer and subsequently, curing it at T=60 °C. The image of the fabricated CAPSENSAR and its integration on the gripper palm are shown in Supp. Fig. 7h and Supp. Fig. 7h Inset 3 respectively. The CAPSENSAR utilizes cost effective materials, follows a mask less fabrication technique with customizable electrode design, avoids wastage of printable ink due to printed electrodes and circuitry, involves easy fabrication steps-inkjet printing of graphene electrodes on PI, spin coating of Eco flex on BE and spray coating of PVA on TE and fast printing with print throughput of 15 min and printing rate 20 mm s^{-1}, makes it commercially viable for rapid production and cost-effective manufacturing.

Supplementary Discussion 4: Construction of the COGBOT

The different parts of the COGBOT comprising of the (i) concealed casing of spur gear arrangement, (ii) upper and lower arms and (iii) outer frame of the palms of the robotic gripper were fabricated by 3D printing the Acrylonitrile Butadiene Styrene (ABS) filament using Ultimaker 2+ 3D printer and assembled by using nut-bolts shown in Supp. Fig. 8. A 2 mm thick PDMS layer was incorporated within the outer frame of each gripper palm of the robotic arm using nut-bolts. This PDMS layer facilitates the gripper palms to conformably grip the target object, thereby reducing the probability of slippage and damage of object and thus, makes the COGBOT suitable for handling the delicate object. The commercially brought gears, wheel and servos and other components were assembled using screws. The

as-fabricated CAPSENSARs were attached with the PDMS layer of each of the two gripper palms using an eco-flex layer.

Supplementary Discussion 5: Characterization of CAPSENSAR

The separate experimental set up was designed to characterize the (i, j) elementary sensor unit of the CAPSENSAR, and estimate the face landscape using sensor array. The COGBOT was tested with a pair of CAPSENSARs attached on either palm of the robotic gripper where the data acquisition and implementation of sequential task were performed in Arduino mega microcontroller (MC) housed inside the controlling unit.

Section 5.1: Experimental set up

The investigation of the elementary sensor unit incorporates the characterization of sensor unit as proximity and pressure sensors. The proximity sensing of the CAPSENSAR was characterized using a custom-built laboratory set up consisting of a wooden base and an elevated cantilever having a series of wheels, through which the distance *z* of the object from the device can be regulated by a wheel and thread arrangement using a battery powered 3V-DC mini motor as shown in Supp. Fig. 9a. The direction of rotation of the dc motor was controlled using a bidirectional switch. The counter clockwise rotation of dc motor driven wheel 1 decreased the proximal distance *z* while its clockwise rotation increased *z*. The *z* of the object from the device was measured using a ruler fixed perpendicularly to the plane of device. The object was suspended by a thread from wheel 3 and aligned at the center of the plane of the device. The experiment was performed in a confined room so that there was minimal variation in spatial position of the object under test.

Section 5.2: Circuit for Electrical Measurements

The characterization of all the (i, j) senor units were performed at the respective sites on the device where the jth BE overlaps with the ith TE of the CAPSENSAR. The sensor unit was characterized by connecting the TE and the BE terminals to the Keithley 595 capacitance meter instrument. The face landscape estimation was performed by exposing the full area of the CAPSENSAR to the target object face where each $(i\{=1,2,3,4,5\}\times j\{=1,2,3,4\})$ sensor array separately measures the output capacitance in presence of object at a known distance *z*= 10 mm to generates a capacitive impression of this object face. The CAPSENSAR was connected to the MC through an 8:1 Multiplexer (SN74LS151N) MUX. The data acquisition (or recording of $\left.C_{out}^{i,j}\right]^{pre}$) for all (i, j) elementary sensor units of the CAPSENSAR were performed by connecting the (i=1,2,3,4,5) terminal of TE to the five input pins of each MUX, while the (j=1,2,3,4) terminals of BE and the output pins of MUX were connected to the analog pins of the MC (Supp. Fig. 9b). The MC was preprogramed to measure the output capacitance of each (i, j) sensor units through selection

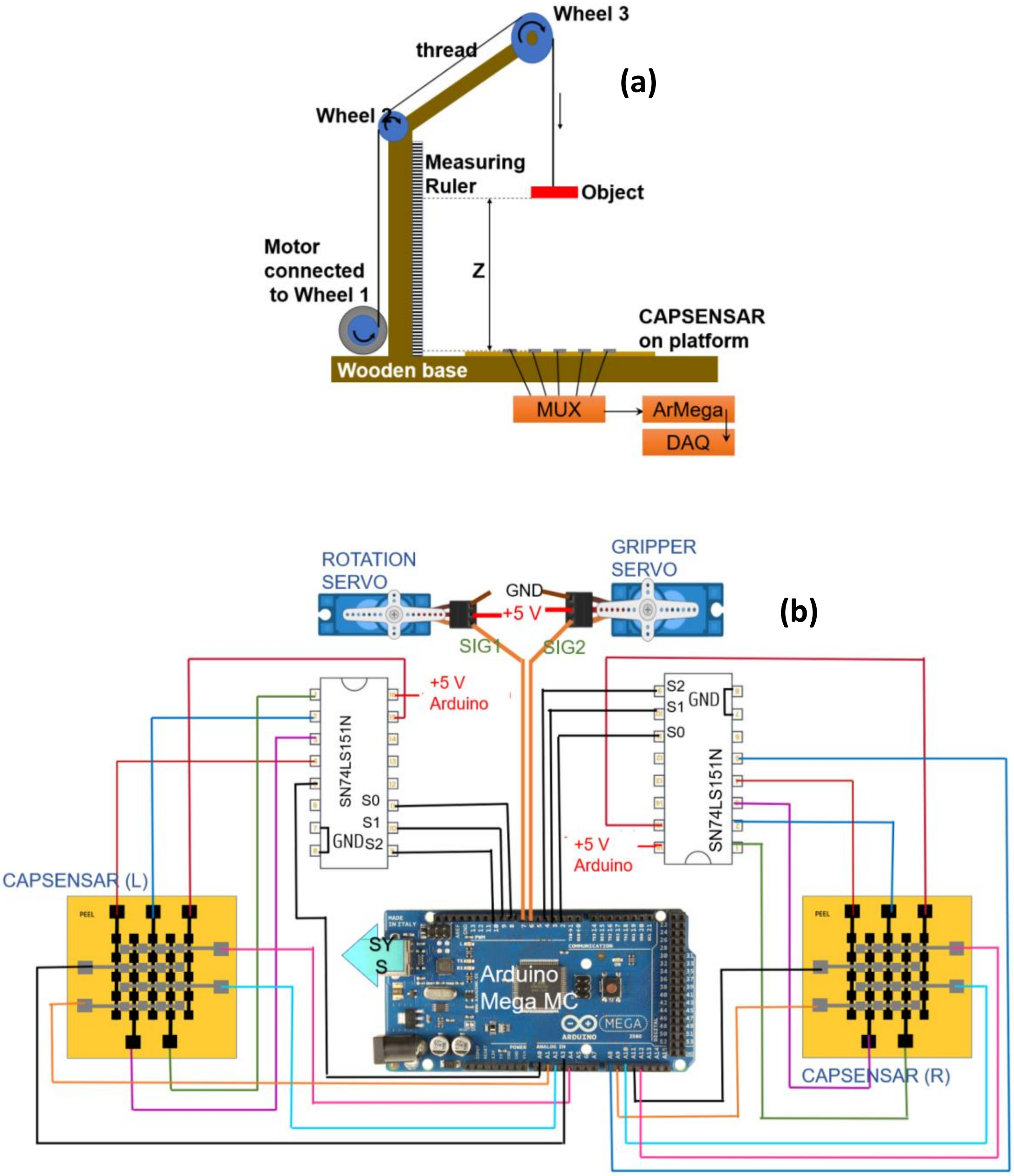

Supplementary Figure 9: Experimental test set up for CAPSENSAR and COGBOT. a. Schematic representation of set-up for electrical characterization of CAPSENSAR and **b.** Circuit diagram for acquiring the capacitive outputs of the different (i, j) elementary sensor units of the CAPSENSAR and for the operation of COGBOT.

pins (S0, S1 and S2) of the MUX. The data acquisition for $C_{out}^{i,j}\Big]^{pre}$ of each (i, j) sensor units of the CAPSENSAR were implemented using parallax data acquisition (PLX-DAQ) software and the data were stored in the computer and monitored on the screen. The pressure sensor units were characterised by Mark-10 Force gauge with G1010-2 Jacob Chuck pin Vise grip with 3 mm diameter, where pressure was separately exerted on each (i, j) senor unit of the

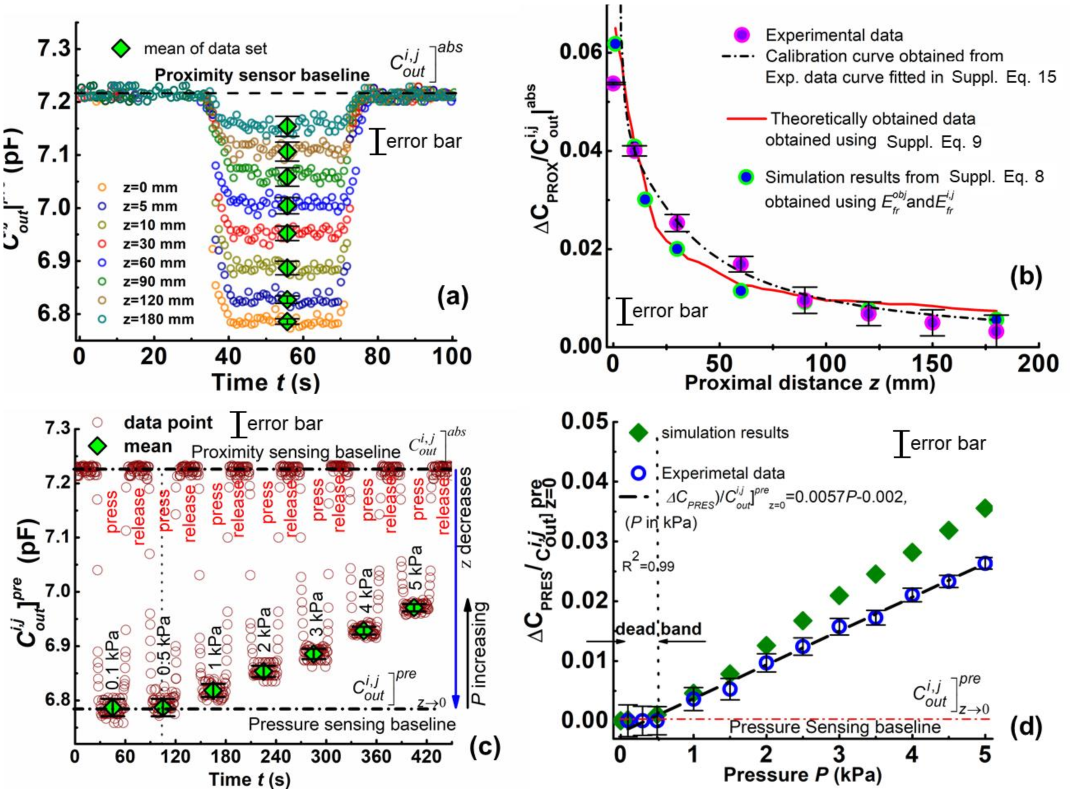

Supplementary Figure 10: Electrical characterization of proximity and pressure sensing unit. a. Dynamic measurement of output capacitance $\left.C_{\text{out}}^{\text{i,j}}\right]^{\text{pre}}$ with time for an approaching object at different distances *z*= 0,5,10, 30, 60, 90, 120, and 150 mm, recorded by the (i, j) sensor unit of CAPSENSAR, The mean $\left.C_{\text{out}}^{\text{i,j}}\right]^{\text{pre}}$ for different *z*-data sets (acquired when the object approaches the device during dynamical measurement) are denoted by green rhombus points with electronic fluctuation represented by error bars. **b.** Normalized change in output capacitance $\Delta C_{\text{PROX}} \Big/ \left.C_{\text{out}}^{\text{i,j}}\right]^{\text{abs}}$ vs. proximity distance *z* calibration curve of a (i, j) sensor unit for an object of stainless steel, relative to the results obtained from theoretical and simulation studies, **c.** Dynamic measurement of output capacitance $\left.C_{\text{out}}^{\text{i,j}}\right]^{\text{pre}}$ with time for different applied pressures *P*= 0.1, 0.5, 1,2,3,4 and 5 kPa, showing alternative pressure and release cycles when the object was approached onto the (i, j) sensor unit from a distance of *z*=180 mm for each cycle. The green rhombus points denote the mean of the data points acquired when the object undergoes different pressure cycles. The electronic fluctuation is shown by error bars. and **d.** Normalized change in output capacitance $\Delta C_{\text{PRESS}} \Big/ \left.C_{\text{out}}^{\text{i,j}}\right]^{\text{pre}}_{z\to 0}$ vs. Pressure *P* calibration curve of (i, j) sensor unit showing a dead band in the range 0-0.5 kPa and its comparison with simulation results. The errors in measurements form 20 sensor units were represented by error bars.

CAPSENSAR and the data were recorded for respective its (i, j) senor units. The $\left.C_{out}^{i,j}\right]^{pre}$ of all (i, j) sensor units of the CAPSENSAR under applied pressure were recorded using the MUX and MC as described earlier. The COGBOT was tested with the pair of CAPSENSAR connected to the MC through separate 8:1 MUX as shown in Supp. Fig. 9b. The rotation and the gripping servos communicate with the MC through Signal 1 (SIG1) and Signal 2 (SIG2) respectively. The Arduino Mega board is powered by the computer.

Section 5.3: Characterization of elemenary sensor units

We characterize the elementary sensor units (i, j) of the CAPSENSAR as proximity and pressure sensor units. The capacitive outcomes from all the (i, j) sensor units in the CAPSENSAR constitute capacitive impressions in the form of matrix which was further transformed into *z*-matrices and *P*- matrices when the CAPSENSAR was operated in proximity and pressure sensing mode respectively. The *z*- matrices and the *P*- matrices of the capacitive impressions yield the *z*-contour and the *P*- contour plots respectively. The *z*-contour was used for contactless three-dimensional face landscape estimation of the exposed face of the target object while the *P*-contour was used to ensure reliable gripping of the object. All electrical characterizations were performed by positively biasing TE relative to BE. The experimental set-up for device testing is described in Supp. Discussion 5.

The electrical characterization of all (i, j) sensor units of the CAPSENSAR was performed to investigate the response of that sensor unit for (a) an approaching object at a distance *z* along the normal to that (i, j) sensor unit and the (b) pressure exerted on that (i, j) sensor unit. Since each sensor unit of the CAPSENSAR spans over an area of 3×3 mm^2, the unit was tested using a stainless-steel cylinder of diameter ~*a*=3 mm and height 10 mm to reduce the effects of interfering field lines from large objects.

Section 5.3.1: Proximity sensor units

All (i, j) sensor units of the CAPSENSAR were investigated for proximity sensing with different proximal distances *z* in the range of 0-180 mm. The dynamic measurements of output capacitance in present of object $\left.C_{\text{out}}^{\text{i,j}}\right]^{\text{pre}}$ for the (i, j) sensor unit were performed when the target object was kept at different distances *z* from that sensor unit as shown in the Supp. Fig. 10a. The data were recorded at a time interval of 200 ms over a total sampling time of 100 s when the CAPSENSAR was kept flat on the testing bed. The mean ($\text{Mean}\left|\left.C_{\text{out}}^{\text{i,j}}\right]^{\text{pre}}\right|$) for different *z* were calculated from the dynamic data and the corresponding error (e_{fluc}) due to electronic fluctuations were represented as error bars as shown in Supp. Fig. 10a. The dynamic $\left.C_{\text{out}}^{\text{i,j}}\right]^{\text{pre}}$ for a given (i, j) sensor unit were found to decrease from the proximity sensor baseline $\left.C_{\text{out}}^{\text{i,j}}\right]^{\text{abs}}$ on exposure of the object to that sensor unit. The average instability error δ_{stab} for all sensor unit was calculated using the Eq. $\delta_{\text{stab}} = \dfrac{e_{\text{fluc}}}{\text{Mean}\left|\left.C_{\text{out}}^{\text{i,j}}\right]^{\text{pre}}\right|} \times \mathbf{100\%}$ and found to be δ_{stab} =3.5%. The steady fringing field distribution due to the uniform thickness of printed electrodes and low leakage current generation in the non-porous, highly thermal resistant and electrically insulated PI layer help to achieve the high stability in the performances of the sensor unit. The response time of the proximity sensor unit was graphically determined from Supp. Fig. 10a to be 0.3 s. The low response time may be attributed to electrostatic working mechanism of the sensor and enables high switching rate for rapid response sensors for fast generation of capacitive impressions of the exposed

face of the object.

Different (i, j) sensor units in the CAPSENSAR were investigated and $\left.C_{\text{out}}^{\text{i,j}}\right]^{\text{pre}}$ were recorded at various z in the range=0-180 mm for all sensor units of sample size 20. The normalized change($\Delta C_{\text{PROX}} / \left.C_{\text{out}}^{\text{i,j}}\right]^{\text{abs}}$) in output capacitance $\left.C_{\text{out}}^{\text{i,j}}\right]^{\text{pre}}$ for different z relative to $\left.C_{\text{out}}^{\text{i,j}}\right]^{\text{abs}}$ were calculated using Supp. Eq. (9) and the mean of respective data set (of 20 data points) for different z were plotted in Supp. Fig. 10b. The mean $\Delta C_{\text{PROX}} / \left.C_{\text{out}}^{\text{i,j}}\right]^{\text{abs}}$ for the (i, j) sensor unit increases as the z decreases when the object approaches the plane of that sensor unit and is attributed to the generation of increased change in intrinsic fringing field $\Delta E_{\text{fr}}^{\text{i,j}} = E_{\text{fr}}^{\text{i,j}} - E_{\text{fr}}^{\text{obj}}$ at that sensor unit due to presence of the object at reduced z as evident from Supp. Eq. (8) and described in Supp. Disc. 2.1. This variation in $\Delta C_{\text{PROX}} / \left.C_{\text{out}}^{\text{i,j}}\right]^{\text{abs}}$ with z was found to be sharp in the range 0-30 mm beyond which the variation gradually reduced. Since the $\Delta C_{\text{PROX}} / \left.C_{\text{out}}^{\text{i,j}}\right]^{\text{abs}} - z$ curve saturated beyond z=120 mm the dynamic range of the proximity sensor was considered to be z=0-120 mm. The calibration curve for each (i, j) sensor unit was obtained by fitting the experimental data in the non-linear equation as:

$$\frac{\Delta C_{\text{PROX}}}{\left.C_{\text{out}}^{\text{i,j}}\right]^{\text{abs}}} = \frac{M_1}{z.\left[M_2 - M_3 \times \tan^{-1}\left(\dfrac{M_4}{z\sqrt{M_5 + 4z^2}}\right)\right]\left[1 + 4\left(\dfrac{z}{\sqrt{z^2 + 36}}\right) + 4\left(\dfrac{z}{\sqrt{z^2 + 72}}\right)\right]} \quad \text{...........(Supp. Eq. 15)}$$

where M_1=25.7 V, M_2=343.3 V m^{-1}, M_3=40.1 V m^{-1}, M_4 =28.5×10^{7} m^{-2} and M_5=4×10^{10} m^{-2} for stainless steel object as shown in Supp. Fig. 10b. The Supp. Eq. (15) obey theoretically calculated Supp. Eq. (9) and validated with simulation results in Supp. Fig. 10b. The sensitivity of the proximity sensor unit was determined from the slope of the $\Delta C_{\text{PROX}} / \left.C_{\text{out}}^{\text{i,j}}\right]^{\text{abs}} - z$ curve in Supp. Fig. 10b and found to be 0.012 mm^{-1} in the range z=0-30 mm. The z-resolution $\Re z$ of the device within z< 30 mm was obtained from the Supp. Fig. 10a and b to be 0.091 mm. However, at higher distances z> 120 mm the z-resolution was found to be in the order of tens of mm. The high stability and excellent z-resolution in the range 0-30 mm make the device suitable for use in surface landscape detection within z=30 mm.

Section 5.3.2: Pressure sensor unit

The electrical characterization of the (i, j) sensor unit in response to pressure was performed under the same biasing conditions at different pressures (P) in the range 0.1-5 kPa. The dynamic measurement on the (i, j) sensor unit was performed with six different P=0.1 kPa, 0.5 kPa, 1 kPa, 2 kPa, 3 kPa, 4 kPa and 5 kPa. The $\left.C_{\text{out}}^{\text{i,j}}\right]^{\text{pre}}$ data were recorded at an interval of 200 ms when the object was subjected different pressures under alternative pressure and release cycles of 30 s each, spanning over a total time period of 390 s as shown in Supp. Fig. 10c. During dynamic data acquisition,

the object was descended from the proximity sensing baseline $\left.C_{\text{out}}^{\text{i,j}}\right]^{\text{abs}}$ =7.19 pF onto the (i, j) sensor unit until it reaches the pressure sensing baseline $\left.C_{\text{out}}^{\text{i,j}}\right]_{z\to 0}^{\text{pre}}$ =6.78 pF at z=0 mm. The response time and instability error δ_{stab} of pressure sensor unit was graphically obtained from Supp. Fig. 10c to be 0.4 s and 5.3% respectively.

The experiment was repeated for all (i, j) sensor units in the array and the respective $\Delta C_{\text{PRES}} \Big/ \left.C_{\text{out}}^{\text{i,j}}\right]_{z\to 0}^{\text{pre}}$ for all (i, j) sensor units at different P were calculated using the expression $\Delta C_{\text{PRES}} = \left.C_{\text{out}}^{\text{i,j}}\right]^{\text{pre}} - \left.C_{\text{out}}^{\text{i,j}}\right]_{z\to 0}^{\text{pre}}$. The mean $\Delta C_{\text{PRES}} \Big/ \left.C_{\text{out}}^{\text{i,j}}\right]_{z\to 0}^{\text{pre}}$ for the data set obtained for all (i, j) sensor units at each P is determined and $\Delta C_{\text{PRES}} \Big/ \left.C_{\text{out}}^{\text{i,j}}\right]_{z\to 0}^{\text{pre}}$ is plotted in Supp. Fig. 10d. The errors in measurements were represented as error bars. The (i, j) pressure sensor units suffer a dead band region between 0-0.5 kPa where the sensor unit showed no variation in $\Delta C_{\text{PRES}} \Big/ \left.C_{\text{out}}^{\text{i,j}}\right]_{z\to 0}^{\text{pre}}$. This is attributed to the local stiffness of the 30 µm thick PI sheet bearing the printed electrodes, constituting the proximity sensor array in the device. This stiffness of the PI sheet is unable to produce measurable change in output capacitance $\left.\text{C}_{\text{out}}^{\text{i,j}}\right]^{\text{pre}}$ and thus limits the detection of very low applied pressure in the range 0-0.5 kPa termed as the dead band region. However, beyond P>0.5 kPa, the $\Delta C_{\text{PRES}} \Big/ \left.C_{\text{out}}^{\text{i,j}}\right]_{z\to 0}^{\text{pre}}$ increases with P in the dynamic range 0.5-5 kPa. The experiment was terminated at P=5 kPa to prevent damage of the graphene printed electrodes. The calibration curve $\Delta C_{\text{PRES}} \Big/ \left.C_{\text{out}}^{\text{i,j}}\right]_{z\to 0}^{\text{pre}}$ - P for the (i, j) sensor unit is obtained by fitting the experimental data in Supp. Eq. (16) as:

$$\frac{\Delta C_{\text{PRES}}}{\left.C_{\text{out}}^{\text{i,j}}\right]_{z\to 0}^{\text{pre}}} = W_1 \times \text{P-} W_2 \quad \text{(Supp. Eq. 16)}$$

where, W_1 =0.0057 kPa^{-1} and W_2 =0.002 for stainless steel object. The increase in $\Delta C_{\text{PRES}} \Big/ \left.C_{\text{out}}^{\text{i,j}}\right]_{z\to 0}^{\text{pre}}$ with P in dynamic range is attributed to reduction in Ecoflex thickness δ at increased pressure P. Supp. Eq. (16) obey the theoretically derived Supp. Eq. (13) and validated with simulation result as shown in Supp. Fig. 10d. The sensitivity of the (i, j) sensor unit was obtained from the slope of the calibration curve as 0.006 kPa^{-1}. Good response time and low instability error of the sensor unit facilitates fast and precise estimation of the optimum gripping force during gripping and also execute reliable and damage free gripping of the target object by COGBOT.

Section 5.4: Detection of face landscape using CAPSENSAR

In this section, the proximity sensor array was utilized for the determination of three-dimensional face landscape of the target object in terms of capacitive impression as recorded by respective (i, j) elementary sensor units of

CAPSENSAR. The $\left.C_{\text{out}}^{\text{i,j}}\right]^{\text{pre}}$ recorded for all (i, j) elementary sensor units of the CAPSENSAR were compounded for determining the capacitive impression of the exposed object face. The demonstration for landscape estimation of the object face was performed using stainless steel objects of various geometrical shapes - sphere, cone and a disc with dimensions as given in Supplementary Table 1. The demonstration was performed by aligning the object at the center of the CAPSENSAR plane and kept stationery at a z=10 mm. The electronically recorded $\left.C_{\text{out}}^{\text{i,j}}\right]^{\text{pre}}$ for respective (i, j) sensor units were converted to the $\left.C_{\text{out}}^{\text{i,j}}\right]^{\text{pre}}$ matrix through gridding and subsequently plotted as contour representation of $\left.C_{\text{out}}^{\text{i,j}}\right]^{\text{pre}}$ using the software Origin 8.5 to obtain a capacitive impression of the object face under exposure to CAPSENSAR. These capacitive impressions were used for the estimation of the three-dimensional (3D) face landscape of the different objects such as sphere, cone and disc as shown in Supp. Fig. 11a, b and c respectively. The capacitive impression for each object was obtained by the virtue of variation in $\left.C_{\text{out}}^{\text{i,j}}\right]^{\text{pre}}$ of respective (i, j) sensor units in response to the 3D profile of that object face exposed to the CAPSENSAR. The 3D profile of the object face produces segment-wise change in the $E_{\text{fr}}^{\text{obj}}$ and hence $(\Delta E_{\text{fr}}^{\text{i,j}} = E_{\text{fr}}^{\text{i,j}} - E_{\text{fr}}^{\text{obj}})$ captured by the corresponding perpendicularly projected (i, j) sensor unit to undergo respective change in (i, j) elements of the $\left.C_{\text{out}}^{\text{i,j}}\right]^{\text{pre}}$ matrix as obtained in Supp. Discussion 2.2. The dimensions and landscape of the object face can be estimated when the (i, j) elements of the $\left.C_{\text{out}}^{\text{i,j}}\right]^{\text{pre}}$ matrix (as shown in Supp. Fig. 11a ,b and c were transformed into its corresponding $z_{\text{i,j}}$ to yield the z-matrix using the relation Supp. Eq. (15). The $z_{\text{i,j}}$ denotes the perpendicularly projective proximal distance of the object from the $(i, j)^{\text{th}}$ sensor unit of the CAPSENSAR. The $z_{\text{i,j}}$ elements constituting the z-matrix of a given object face were gridded in 5x4 matrix and illustrated as 3D z-contour plot representation using Origin 8.5 software for sphere, cone and disc as shown in Supp. Fig. 11d, e and f respectively. Since the elements of the z-matrix denote the segment-wise normal proximal distances z of the object from the respective (i, j) sensor units, it provides an estimate about the dimensions of the object face as well as the spatial variation in object landscape. The two-dimensional profile of the exposed faces of different the objects-(i) sphere, (ii) cone and (iii) disc along their (1) x-and (2) y-dimensions are shown in Supp. Fig. 11(g, h), (i, j) and (k, l) respectively. The accuracy in measurement of object

Supplementary Table 1

Dimensions of Objects of different shapes

Shape of Objects	Dimensions
Sphere	Diameter 20 mm

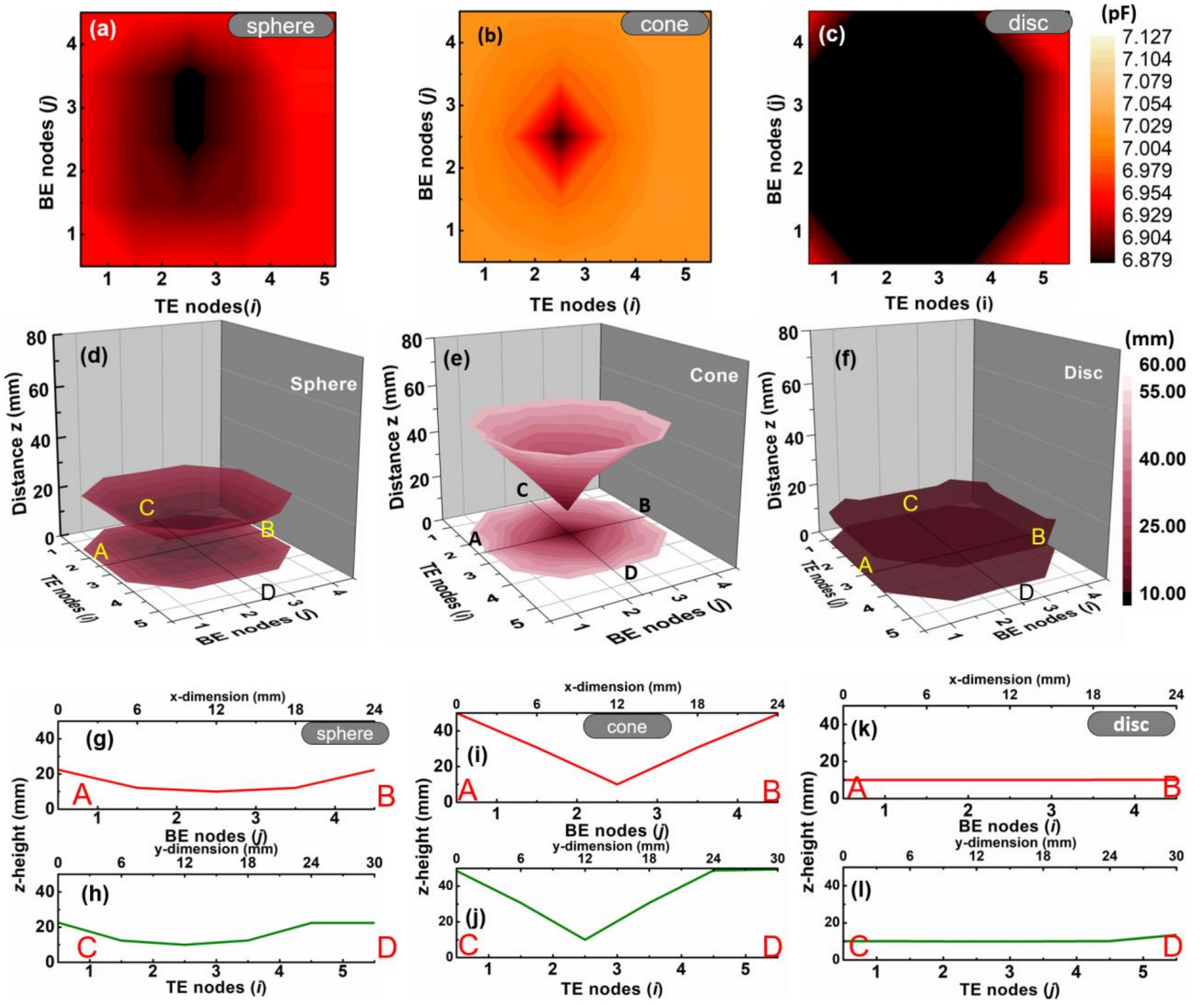

Supplementary Figure 11: Determination of face landscape and estimation of object shape and size using CAPSENSAR. Contour plots showing capacitive impressions of exposed faces of **a.** sphere, **b.** cone and **c.** disc and corresponding three-dimensional *z*-contour profiles of exposed faces of d. sphere, **e.** cone and **f.** disc. Two-dimensional *z*-contour profile along points A and B in *x*-direction and along points C and D in *y*-direction for objects sphere (**g., h.**), cone **(i., j.**) and disc (**k., l.**) respectively, and used for size estimation

Cone	Basal diameter 20 mm, height 30 mm
Disc	Diameter 20 mm and height 2 mm

dimension in the *x*-*y* plane was obtained to be approx. 80%. The *x*-*y* flatness resolution depends on the dimension of electrode size and the interelectrode distance $a, \zeta = 3$ mm. Since the accuracy in the *x*-*y* measurement was 80%, the flatness resolution $\Re$ at a distance *z* from the device is 3+(0.2 *z*). The flatness resolution of the device deteriorates as the object was placed further away from the device and was attributed to the diverging fringing electric field of the device. The *z*-contour plot was utilized in ascertaining the fittest gripping face of the target object prior to gripping using COGBOT.

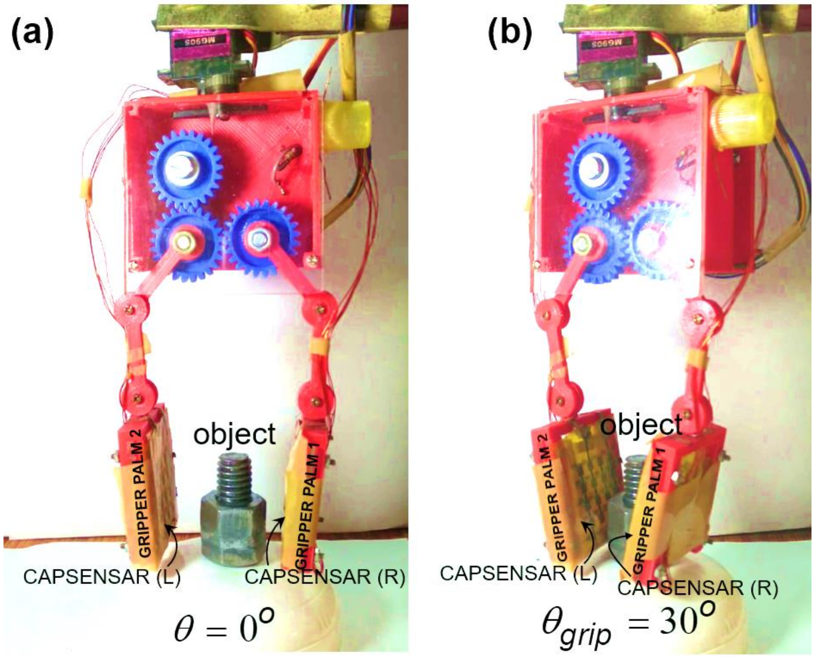

Supplementary Figure 12: Determination of optimum gripping pressure P_{grip} on opposite pair of flattest faces of the object. Image of the COGBOT and the target object between the palms at the **a.** initial position θ=0° where pair of palms with CAPSENSAR was aligned parallel to Face 1 and **b.** at the gripping alignment angle θ_{grip}=30º (in this case) where the COGBOT ascertains the optimum gripping pressure P_{grip} with pre-loaded gentle touch of P=0.5 kPa

Supplementary Discussion 6: Secure gripping and Prevention of slippage

The operation of the COGBOT commences after initialization as shown in Supp. Fig 12a. When the opposite pair of flattest faces on the object was determined at $\theta = \theta_{grip}$, the COGBOT palms with CAPSENSAR (L) and CAPSENSAR (R) converged on the object to grip the target object to determine the optimum gripping pressure $P_{\rm grip}$ to ensure safe and effective gripping (Supp. Fig 12b). For this, the palms touch the opposite faces of the object with pre-loaded P=0.5 kPa when the COGBOT executes a number of iterative processes to obtain $P_{\rm grip}$ and subsequent revise it to $P_{\rm grip}^{\rm new}$ during slippage or deformation. Initially the CAPSENSAR (L, R) generated separate *P*-contour plots from their respective capacitive matrices for opposite gripping faces of the object. Supp. Fig. 13a show the *P*-contour plot of one of the opposite gripping faces of the object at θ_{grip}=30º. Under gentle touch state *P*=0.5 kPa, the estimation of the null matrices $N_{\rm L}$ and $N_{\rm R}$ with CAPSENSAR (L) and CAPSENSAR (R) was obtained using the *P*-matrix plot as shown in Supp. Fig. 13b. The respective area of contact $A_{\rm L}$ and $A_{\rm R}$ on opposite gripping faces of the object corresponding to the $N_{\rm L}$ and $N_{\rm R}$ could be accurately determined for object orientation as shown in Supp. Fig. 13c. Since the area of each pressure sensing unit i.e. $A_{\rm unit}$ = 36 mm^2, the flattest areas of contact for gripping, as obtained for respective CAPSENSAR (L, R), were determined to be $A_{\rm L} = N_{\rm L} \times A_{\rm unit}$ and $A_{\rm R} = N_{\rm R} \times A_{\rm unit}$ respectively and stored in the MC. The critical gripping pressure P_{crit} required for deformation and slippage free gripping was determined to be:

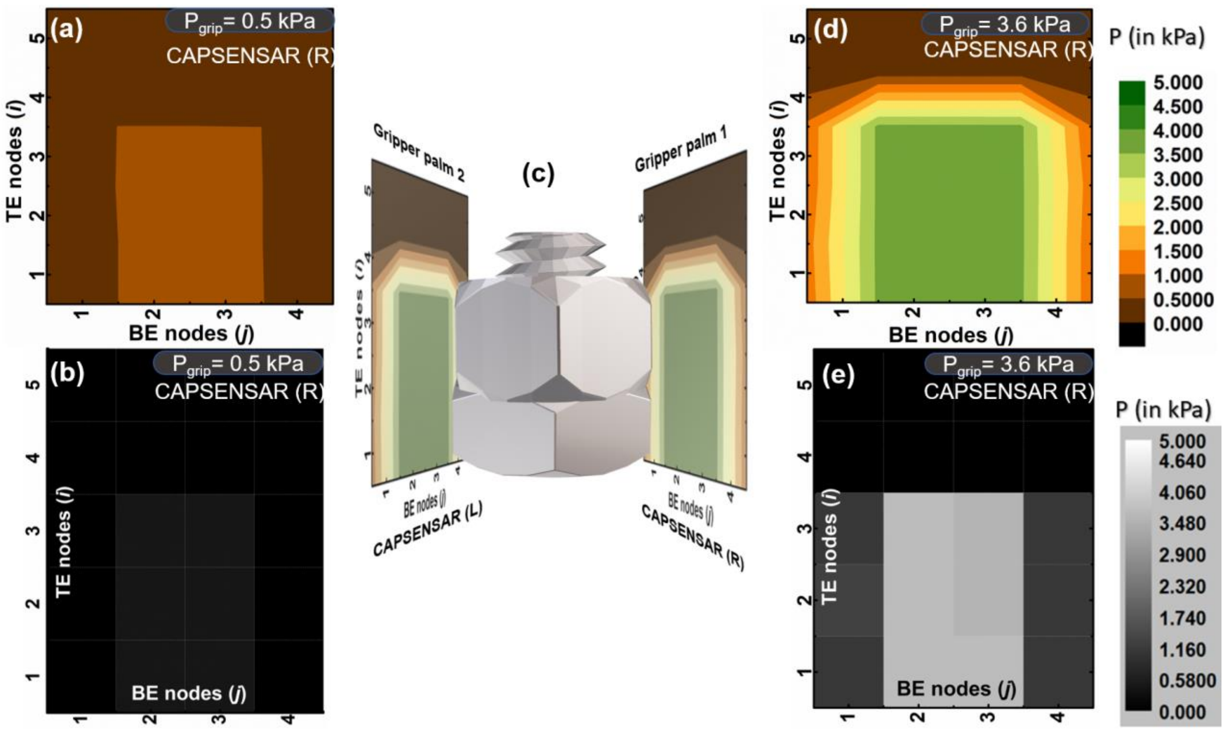

Supplementary Figure 13: Secure gripping of object with optimum gripping pressure P_{grip} at the gripping alignment angle θ_{grip}=30°. For CAPSENSAR (R), Gridded *P*-contour representation **a.** and *P*-matrix plot **b.** of the fittest pair of opposite flattest faces at θ_{grip}=30° when gentle touch *P*=0.5 kPa was applied on the pair of faces to estimate the optimum gripping pressure P_{grip} required for effective gripping**, c.** Orientation of the object between the gripper palms 1 and 2 as confirmed by the similarity in the null elements N_R pattern obtained from the *P*-matrix plots in **b.**, and Gridded *P*-contour representation **d.** and *P*-matrix plot **e.** of the same faces at θ_{grip}=30° when the object was grasped between the gripping palms 1,2 with P_{grip} =3.6 kPa, ensuring safe and secure gripping without slippage. The brown region in **a.** denotes the region of contact of CAPSENSAR with the object under gentle touch while the green region in **d.** denotes the area of contact at increased pressure P_{grip} =3.6 kPa. The dark grey regions in **b.** denotes the positions of null elements in the *P*-matrix under gentle touch while the grey regions in **e.** denotes the same under P_{grip}= 3.6 kPa.

$$P_{crit} = \frac{mg}{Min(\Lambda_L, \Lambda_R)} \quad \text{……… Supp. Eq. (17).,}$$

here *m* and *g* denote the mass of the object and gravitational acceleration respectively. The value of P_{crit} is calculated in MC and thereafter instructs the grip controlling servo motor to grip the object with a gripping pressure of P_{grip}=1.2 P_{crit}, providing an additional tolerance pressure of 0.2 P_{crit} to ensure successful and reliable grip. In this work using the custom-made object P_{grip} was graphically calculated from Supp. Fig. 13b to be 3.6 kPa for N_L, N_R =6. This value of P_{grip}=3.6 kPa was confirmed from the *P*-contour and *P*-matrix representations when the object was gripped with optimal pressure P_{grip} as shown in Supp. Fig. 13d and Supp. Fig. 13e respectively.

Since the iterative cycle for the determination of N_L and N_R was continuously updated throughout the entire duration of operation, the Min $|\Lambda_L, \Lambda_R|$ and hence, the values of P_{crit} and P_{grip} were updated in the MC accordingly. Supp. Fig. 14a and Supp. Fig. 14b show the pressure landscapes of CAPSENSAR (L) and (R) respectively in the subsequent iterative cycle under undesirable slippage of the target object. During slippage, the newly recorded N_L, N_R = 5 could

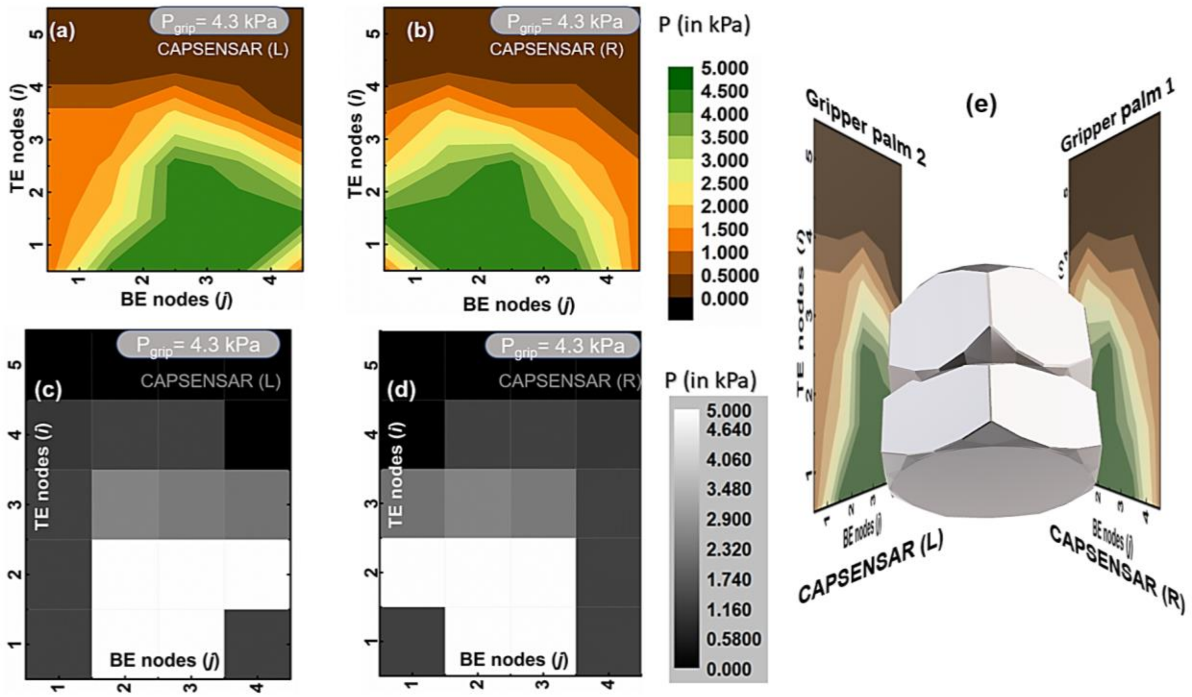

Supplementary Figure 14: **Anti-slippage operation in COGBOT and the determination of new P_{grip} during slippage.** Gridded *P* contour representations for **a.** CAPSENSAR (L) and **b.** CAPSENSAR (R) during slippage of object when the object was grasped by revised P_{grip} =4.3 kPa which was recalculated by considering the updated values of N_L and N_R in subsequent iterative cycles from the newly generated *P*-matrix plots **c.** and **d.** for CAPSENSAR (L) and CAPSENSAR (R) respectively, **e.** Tilted orientation of the object due to slippage which was resisted by increased P_{grip} =4.3 kPa. The green regions in **a.** and **b.** denotes area of contact with the object experiencing slippage and the white regions in **c.** and **d.** denotes the positions of null elements in the *P*-matrix under new P_{grip}= 4.3 kPa

be accurately determined using the *P*-matrix plot as shown in Supp. Fig. 14c and Supp. Fig. 14d respectively. The object orientation during slippage is shown in Supp. Figure 14e. The updated areas of contact Λ_L and Λ_R with the CAPSENSAR (L, R) decreased which instructed the MC of the COGBOT to increase the $P_{\text{grip}}^{\text{new}}\left(>P_{\text{grip}}\right)=\dfrac{mg}{Min(\Lambda_{\text{L}}^{\text{new}},\Lambda_{\text{R}}^{\text{new}})}$ =4.3 kPa to resists slippage as shown in Supplementary Table 2. The object suffered a second slippage under rotational motion between the palms when subsequent iterative processes prevented its. The *P*-contour plot and the *P*-matrix plot for the second slippage is given in Supp. Fig. 15a, b) and Supp. Fig. 15c, d) respectively for the orientation as shown in Supp. Fig. 15e. The COGBOT prevented slippage by the application of newly calculated $P_{\text{grip}}^{\text{new}}$ =5 kPa.

Supplementary Table 2

Calculation of P_{grip} at different steps in anti-slippage operation of object

State of Gripping		Figure No.	No. of null elements (N) from P- derivative matrix at $\theta=\theta_{grip}$	Flat area on the fittest pair of opposite gripping faces at $\theta=\theta_{grip}$	Calculation of $\lvert P_{grip} \rvert_{L,R}$
Slipped of object due to misalignment of the Palm(L) and Palm(R)	Palm(L)	Supp. Fig. 14 c	$N_L=5$	$\Lambda_L=N_L\times\Lambda_{unit}$ $=5\times36=180$ mm^2	4.3 kPa
	Palm(R)	Supp. Fig. 14 d	$N_R=5$	$\Lambda_R=N_R\times\Lambda_{unit}$ $=5\times36=180$ mm^2	4.3 kPa
Prevention of slippage	Palm(L)	Supp. Fig. 15 c	$N_L=4$	$\Lambda_L=N_L\times\Lambda_{unit}$ $=4\times36=144$ mm^2	5 kPa
	Palm(R)	Supp. Fig. 15 d	$N_R=4$	$\Lambda_R=N_R\times\Lambda_{unit}$ $=4\times36=144$ mm^2	5 kPa

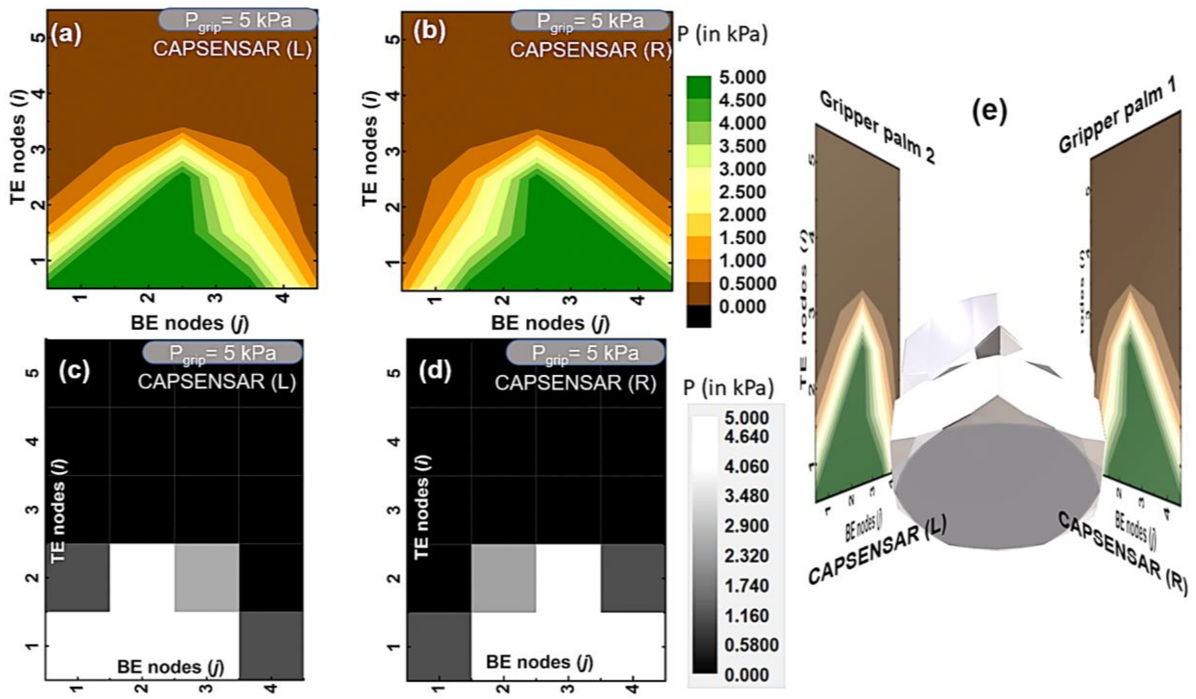

Supplementary Figure 15: Revision of P_{grip} through an iterative cycle to prevent slippage. Gridded P contour representations for **a.** CAPSENSAR (L) and **b.** CAPSENSAR (R) showing slippage motion of the object between the gripper palms. *P*-matrix plots for **c.** CAPSENSAR (L) and **d.** CAPSENSAR (R) obtained through iterative cycles to determine the revised P_{grip} using the number of null matrices N_L and N_R in respective CAPSENSAR, **e.** New orientation of the object due to further slippage which was resisted by revised P_{grip} =5 kPa in the next iterative cycle. The green regions in **a.** and **b.** denote area of contact with the object experiencing slippage and the white regions in **c.** and **d.** denote the positions of null elements in the *P*-matrix under subsequent iterative cycle with P_{grip}= 5 kPa
